# Interoceptive autonomic regulation in typical development and autism spectrum disorder: A computational model integrating multiple physiological systems

**DOI:** 10.1371/journal.pone.0344235

**Published:** 2026-07-15

**Authors:** Ruichen Li, Hao Liu, Yukie Nagai

**Affiliations:** 1 International Research Center for Neurointelligence (WPI-IRCN), The University of Tokyo, Tokyo, Japan; 2 Faculty of Engineering, Chiba University, Chiba, Japan; China Academy of Chinese Medical Sciences Institute of Chinese Materia Medica, CHINA

## Abstract

**Background:**

Interoceptive cardiovascular signals, including heart rate (HR) and blood pressure (BP), arise from coordinated sympathetic (SNS) and parasympathetic (PSNS) regulation and contribute to affective and cognitive processes. Although atypical autonomic nervous system (ANS) modulation has been reported in autism spectrum disorder (ASD), the dynamical structure underlying branch-specific coordination remains insufficiently characterized.

**Objective:**

To estimate latent ANS regulatory structure in typically developing (TD) and ASD individuals using a computational modeling framework.

**Methods:**

A closed-loop computational model integrating cardiovascular, respiratory, and autonomic dynamics was developed. ANS regulation was formalized using three autonomic control modes (coupled reciprocal, coupled nonreciprocal, and uncoupled) and parameterized by effective modulation weights of SNS and PSNS. HR and BP responses to the head-up tilt (HUT) test were simulated, and regulatory surfaces were compared with empirical HR and BP data from TD and ASD groups. Additional simulations under normal respiration, deep respiration, and absence of respiration evaluated mean arterial pressure (MAP) regulation across varying SNS–PSNS activity combinations.

**Results:**

TD individuals exhibited differentiated SNS–PSNS coordination patterns across control modes, whereas ASD individuals showed convergence of SNS–PSNS weight. In TD, HR and BP distributions under coupled reciprocal mode were most consistent with expected physiological responses, characterized by high SNS and low PSNS activity during postural challenge. In ASD, empirical data extended toward regions associated with relatively higher PSNS weighting, suggestive of persistent parasympathetic engagement during postural challenge. Incorporation of deep respiration enhanced MAP reduction during BP recovery, particularly under over-elevated SNS activity.

**Conclusion:**

This study provides a mechanistic, state-space characterization of autonomic coordination in TD and ASD populations, enabling inference of latent autonomic regulation from measurable interoceptive phenotypes and identifying respiration as a model-based regulatory lever that augments cardiovascular stabilization.

## Introduction

The autonomic nervous system (ANS), consisting primarily of the sympathetic and parasympathetic nervous systems (SNS and PSNS), serves as the principal regulatory network governing rhythmic organ functions and modulating interoceptive states to support homeostasis and adaptive responses [1–7]. Cardiovascular and respiratory afferents converge in key central regions such as the brainstem, insula, and anterior cingulate cortex, shaping autonomic output and bodily-state perception [8]. Interoceptive rhythms such as arterial pressure and cardiac cycling activate baroreceptors within the aortic arch and carotid sinus, triggering sympathetic–parasympathetic adjustments to maintain circulatory stability [9–10]. In addition, respiratory activity modulates venous return and stroke volume, generating respiratory sinus arrhythmia that reflects cardiopulmonary coupling [11–12]. Variations in heart rate (HR) and blood pressure (BP) are therefore commonly used as non-invasive indices for assessing sympathetic–parasympathetic balance [13–16].

Growing evidence indicates that atypical interoceptive processing is a hallmark of autism spectrum disorder (ASD), with imprecise interoceptive prediction contributing to sensory atypicality and disrupted emotional-cognitive processing [17–21]. Because interoceptive signals are conveyed through autonomic pathways, altered interoceptive processing may originate from atypical autonomic regulation. Heightened ANS dysfunction in ASD individuals have been reported to affect cardiopulmonary and following social functioning [22–23]. During anxiety, individuals with ASD also exhibit elevated HR alongside reduced electrodermal activity and skin temperature, suggesting atypical SNS–PSNS coordination that may serve as a physiological marker of dysregulation [24]. However, contrasting findings have also emerged that presented typical baseline parasympathetic activity and normal autonomic responses to social stimuli in many autistic children [25], while insufficient sympathetic activation and low physiological arousal were also found in ASD [26], indicating a contrasting profile to excessive sympathetic dominance reported elsewhere. Consequently, the inconsistency in empirical findings on autonomic dynamics constrains the ability to identify differences of interoceptive modulation between typically developing (TD) and ASD individuals, which need for a quantitative and mechanistic framework capable of identifying how differences in autonomic function shape interoceptive cardiovascular responses.

Predictive coding and active inference models conceptualize interoception as generative inference over visceral states, governed by predictions, prediction errors, and precision weighting. Computational formulations reviewed by Petzschner et al. [27] and implemented by Allen et al. [28] enable parameterized characterization of interoceptive regulation, offering a principled framework for comparing interoceptive processing across individuals and populations. However, the existing models focus on a single physiological system or a limited set of interoceptive channels and remain limited in their capacity to capture coordinated regulation across multiple physiological systems. Moreover, their sensitivity to TD-ASD differences has not yet been systematically evaluated. These limitations constrain the explanatory power of current interoceptive models in accounting for differences in interoceptive regulation between TD and ASD populations and highlight the need for computational approaches that integrate multiple physiological systems while providing greater resolution for characterizing group-specific regulatory mechanisms. The objectives of this study are to establish a computational model integrating multiple physiological systems including cardiovascular, respiratory, and autonomic nervous systems. By analyzing the time-varying HR and BP variations, this computational model enables to clarify how ANS functions give rise to differences in HR and BP across TD and ASD populations, and to evaluate whether self-regulation strategies that modulate autonomic output promote recovery of impaired interoceptive cardiovascular rhythms. Achieving these objectives advances mechanistic insight into autonomic contributions to interoceptive regulation and provides a computational foundation for personalized physiological intervention strategies.

## Related works

### Autonomic regulation strategy

Traditionally, autonomic regulation of interoceptive signals has been conceptualized as a reciprocal process, in which SNS and PSNS activities change in a mutually antagonistic manner. Berntson et al. [29] extended this view by introducing the concept of autonomic control modes to characterize a broader repertoire of SNS–PSNS coordination patterns. Specifically, autonomic regulation may operate in (1) a coupled reciprocal mode, in which SNS and PSNS activities change in opposing directions; (2) a coupled nonreciprocal mode, in which the two branches are either coactivated or coinhibited; and (3) an uncoupled mode, in which activity in one branch varies independently of the other. Research on hypoxic challenges by Fukuda et al. [30] and Kollai et al. [31] further supports this framework: severe hypoxia elicits reciprocal ANS control—SNS activation accompanied by PSNS withdrawal—to increase HR and BP for oxygen delivery, whereas mild hypoxia induces SNS–PSNS coactivation that increases vascular resistance while stabilizing HR. Existing evidence [32] suggests that flexibility in ANS control modes likely extends across a range of physiological stimuli and psychological states; however, a systematic characterization of how distinct control modes are recruited across contexts remains lacking.

### Physiological modeling

Multiscale cardiovascular modeling frameworks have been strategically established and applied based on hemodynamic mechanisms and electrical circuit analogies, ranging from 0D lumped-parameter models for global circulation dynamics, to 1D whole-body arterial network models capturing pulse wave propagation, and 3D regional models resolving local blood flow and vessel biomechanics [33–41]. The computational model incorporating respiratory system influences was developed to quantify the effects of different breathing patterns, including normal and deep respiration, on systemic hemodynamics [42–43]. Respiratory-induced oscillations were introduced through a dynamic intrathoracic pressure (ITP) function embedded in BP waveforms, representing thoracic pressure fluctuations and revealing characteristic phase coupling between respiration and cardiovascular dynamics. In addition, autonomic control modeling has evolved from early formulations emphasizing isolated baroreflex or cardiovascular mechanics to more integrative frameworks that incorporate bidirectional sympathetic–parasympathetic pathways, gas exchange regulation, and afferent–efferent coupling mechanisms [44–46]. The present study defines autonomic function using two dimensions: autonomic control modes [29], and the weight levels of the sympathetic and parasympathetic branches. The computational model is initialized using previously reported experimental HR and BP data from TD and ASD groups under external stimulation [47], allowing individualized physiological states to be embedded in the initialization process. Model responses were then examined under systematically varied autonomic parameter settings to characterize state-dependent patterns of autonomic regulation. The resulting responses were evaluated using three-dimensional interoceptive response surfaces to quantify ANS regulatory behavior in terms of control modes and sympathetic–parasympathetic balance.

## Methods

**Fig 1:**
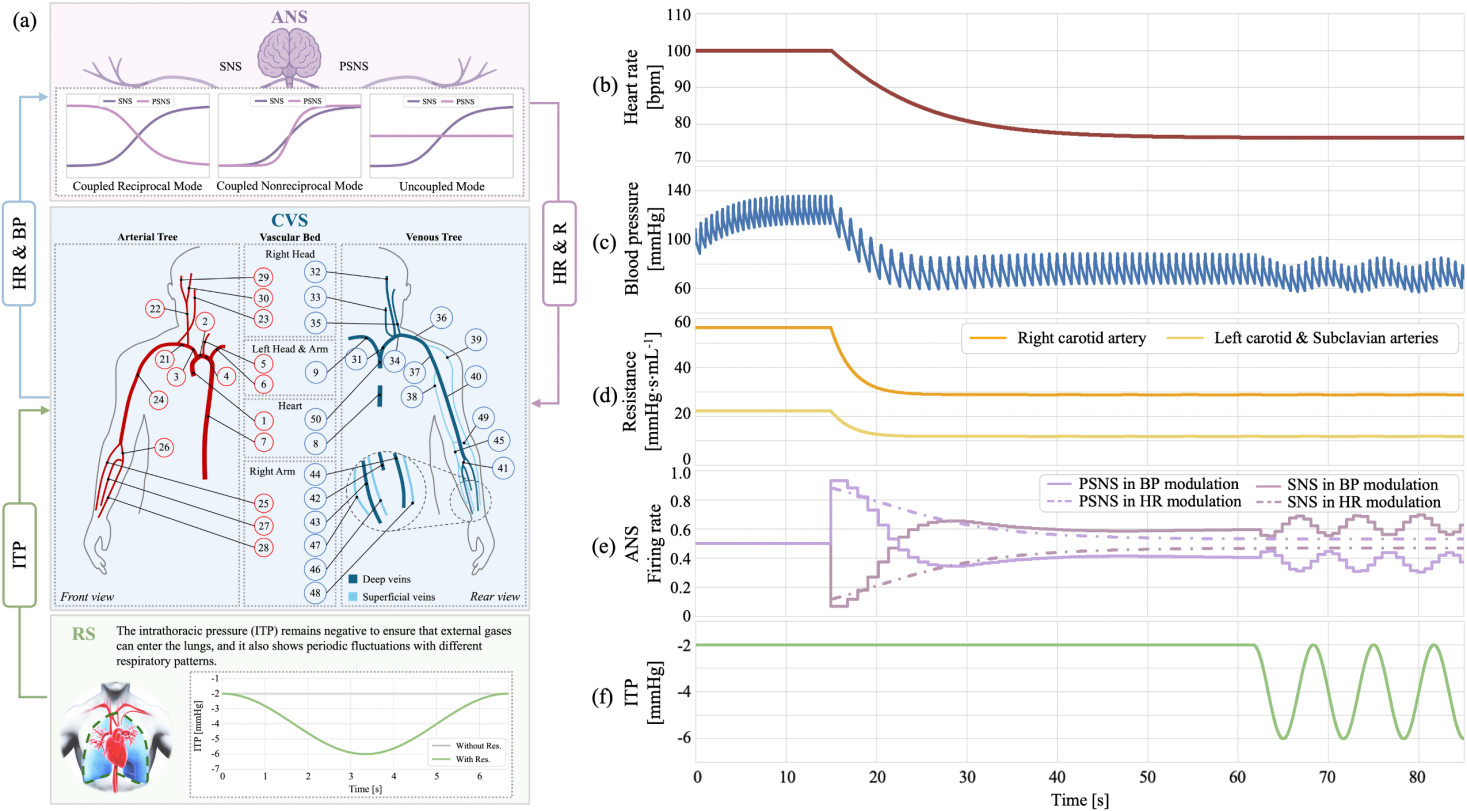
Computational model integrating the cardiovascular, respiratory, and autonomic nervous systems (CVS, RS, and ANS). (a) Schematic overview of the integrated structure of the CVS, RS, and ANS. HR and BP are transmitted to the ANS as fiber afferent activities, while HR itself and blood-flow resistance (R) are modulated by ANS regulatory signals. The R is the key determinant of BP dynamics in the model. ITP waves influence beat-to-beat BP dynamics over longer timescales. (b–f) Representative output signals generated by the model under closed-loop operation, shown across three intentionally designed phases to illustrate the functional contributions of the CVS, ANS, and RS modules rather than to represent distinct biologically occurring physiological stages. Phase I: Elevated initial levels of (b) HR, (c) BP, and (d) blood flow resistance, prescribed to demonstrate CVS-dominated dynamics under a high-load condition. Phase II: HR, BP, and blood flow resistance are gradually restored toward normal physiological ranges under (e) ANS regulatory signals that modulate SNS and PSNS activities, illustrating autonomic feedback regulation. Phase III: ITP oscillations generated by the RS module are introduced, contributing to cyclical variations observed in the BP trace and demonstrating respiration-driven modulation of hemodynamics after autonomic stabilization.

A closed-loop computational model was developed to simulate the dynamic modulation of HR and BP through autonomic regulation by integrating the cardiovascular system (CVS), respiratory system (RS), and autonomic nervous system (ANS). The overall model architecture is shown by the left panel in Fig 1(a). The CVS submodule generates baseline HR and cyclic BP waveforms, which are subsequently modulated by the ANS submodule under different external stimuli and autonomic control modes. The RS submodule influences the CVS-generated BP waveforms in a unidirectional manner by specifying changes in intrathoracic pressure (ITP) associated with different breathing patterns. Building on existing autonomic regulation models, the present study further formalizes ANS function by explicitly decomposing it into two complementary components: autonomic control modes and the weight of the SNS and PSNS branches. The autonomic control modes characterize how SNS and PSNS interact to regulate cardiovascular dynamics, whereas weight captures the branch-specific strength of autonomic modulation.

### Cardiovascular system

The CVS module is responsible for reproducing HR and BP signals that vary in synchrony with the cardiac cycle. The CVS module was built on a closed-loop multiscale hemodynamic modeling framework that integrates hemodynamic model representing major arterial and venous structures, and lumped-parameter model representing cardiac chambers and peripheral arteriovenous interfaces [42–43]. The governing equations are formulated as hemodynamic model approximations of the Navier–Stokes equations along the axial direction of the vessels. The pressure levels within vessels and heart chambers could be defined as:


$$\begin{array}{c} \begin{array}{c} p_{vessel}=\frac{\rho c^{\text{2}}}{k}{\left(\frac{A}{A_{\text{0}}}\right)}^{k}+p_{\text{0}}+p_{ITP}(t), \end{array} \end{array}$$ (1)

$$\begin{array}{c} \begin{array}{c} p_{heart}=E(T,t)\cdot \delta V+S\cdot \frac{dV}{dt}+p_{pc}+p_{ITP}(t), \end{array} \end{array}$$ (2)

where A and $A_{\text{0}}$ are the current and reference cross-sectional areas, respectively, and k is a stiffness exponent characterizing nonlinear wall elasticity. The parameter c is the Moens–Korteweg wave speed, reflecting pulse wave velocity under reference conditions, and $p_{\text{0}}$ is the reference pressure. ρ is the blood density. E(T,t)·δV represents the elastic component, E(T,t) is the time-varying elastance dependent on cardiac cycle T =60/HR and time t, and $\delta V=V-V_{\text{0}}$ is the deviation from reference volume $V_{\text{0}}$. The term S·dV/dt represents viscoelastic component, capturing the viscous dissipation caused by volume changes, where S is the myocardial viscoelastic coefficient, and $p_{pc}$ denotes the pericardial pressure. The time-varying intrathoracic pressure, $p_{ITP}(t)$, is introduced to mimic the respiratory effects on pressure waveforms (see Section 3.2).

Vascular beds are represented using Kirchhoff’s circuit laws, with arterial and venous terminals treated as capacitive nodes connected by blood flow resistance. Volume dynamics are given by:


$$\begin{array}{c} \begin{array}{c} d_{t}V_{a}=Q_{a}-Q_{s}, \end{array} \end{array}$$ (3)

$$\begin{array}{c} \begin{array}{c} d_{t}V_{v}=Q_{s}-Q_{v}, \end{array} \end{array}$$ (4)

$$\begin{array}{c} \begin{array}{c} Q_{s}=\frac{p_{a}-p_{v}}{R}, \end{array} \end{array}$$ (5)

where $V_{a}$ and $V_{v}$ are arterial and venous volumes, and $Q_{a}$, $Q_{v}$, and $Q_{s}$ are inflow from the upstream terminal arteries, the outflow toward the terminal veins, and exchange flow in vascular beds. The $p_{a}$ and $p_{v}$ are arterial and venous pressures. The R denotes the effective flow resistance of the vascular bed, regulating systemic BP levels.

The cardiovascular model combines chamber dynamics, vascular wall mechanics, and blood transport in the arterial and venous trees, enabling unified simulation of the time-varying pressure and flow. By specifying key physiological parameters such as cardiac cycle length, vessel geometry, and peripheral resistance, the model generates stable periodic cardiac dynamics, producing HR rhythms and synchronized BP oscillations. Simulated pressure waveforms in major vessels have been validated against physiological data, confirming that the model reproduces key hemodynamic characteristics of the human CVS.

### Respiratory system

Under regular or controlled breathing conditions, respiration-related intrathoracic pressure (ITP) variations are primarily driven by the rhythmic contraction and relaxation of respiratory muscles. As a result, the temporal profile of ITP typically exhibits smooth and continuous low-frequency fluctuations dominated by a single principal frequency. Compared with the cardiac cycle, the respiratory cycle operates on a substantially longer timescale; therefore, its influence on cardiac chamber pressures and vascular pressures can be regarded as a slowly varying external modulatory component. Based on these physiological characteristics, and in line with previous related work [42–43], the temporal variation of ITP, $p_{ITP}(t)$, is approximated using a simple periodic function:


$$\begin{array}{c} \begin{array}{c} p_{ITP}(t)=a_{\text{0}}+a\cdot \text{cos}(\omega t), \end{array} \end{array}$$ (6)

where $a_{\text{0}}$ denotes the baseline respiration-related BP wave, and the angular frequency ω and amplitude a are specified by the selected respiratory pattern. This term is further incorporated into the analytical formulation of BP to characterize respiration-induced BP fluctuations. The respiratory module provides a phenomenological representation of respiration-related hemodynamic modulation through periodic ITP fluctuations. Therefore, the module is intended to capture the cardiovascular consequences of breathing-related pressure oscillations rather than the full neural–physiological mechanisms underlying cardiorespiratory regulation.

### Autonomic nervous system

#### Autonomic modulation modeling.

The HR signals generated by the CVS module are transmitted to the central nervous system as HR-related afferent fiber activations. BP regulation is modeled based on baroreceptor-mediated mechanism. Three baroreceptors, located in the aortic arch and bilateral carotid sinuses, detect pressure fluctuations in the aorta ($P_{a}$), left and right carotid arteries ($P_{l}$ & $P_{r}$), corresponding to arteries No. 2, 5, and 22 in the CVS module (Fig 1). The baroreceptors are assumed to have identical physiological properties in pressure sensing and signal transduction. Accordingly, the average real-time pressure across the aorta and bilateral carotid arteries is computed and used as the afferent input signal for BP regulation.

Modulation of SNS and PSNS activities was implemented using sigmoid functions to capture their nonlinear responsiveness to HR and BP states in parallel pathways. For each pathway i∈{HR, BP}, the activation levels are defined as:


$$\begin{array}{c} \begin{array}{c} {SNS}_{i}=\frac{\text{1}}{\text{1}+{\left[\frac{F_{aa,i}(t)}{\mu_{i}}\right]}^{\nu_{i,S}}}, \end{array} \end{array}$$ (7)

$$\begin{array}{c} \begin{array}{c} {PSNS}_{i}=\frac{\text{1}}{\text{1}+{\left[\frac{F_{aa,i}(t)}{\mu_{i}}\right]}^{\nu_{i,P}}}, \end{array} \end{array}$$ (8)

where $F_{aa,i}(t)$ denotes time-varying afferent fiber activation during ANS regulation, which can be calculated by $F_{aa,HR}(t)=HR/\text{60}$ representing current HR level in beats per second (bps), and $F_{aa,\;BP}(t)=(P_{a}+P_{l}+P_{r})/\text{3}$ representing average BP across the aortic arch and carotid sinuses. In particular, the initial afferent state at the onset of ANS engagement is denoted as $\overset{\text{0}}{\underset{aa,i}{F}}$, and the final stabilized afferent state after ANS modulation is denoted as $\overset{f}{\underset{aa,i}{F}}$. In contrast, $\mu_{i}$ denotes the baseline HR or BP levels representing the target level under certain internal or external conditions. The parameter $\mu_{HR}$ was determined as the mean of the measured human data, while $\mu_{BP}$ was derived from MAP. MAP was used as the primary index for evaluating BP regulation. The parameters $\nu_{i,S}$ and $\nu_{i,P}$ control the response slopes of the sympathetic and parasympathetic branches, respectively. For each simulation, systolic blood pressure (SBP) and diastolic blood pressure (DBP) were extracted from the model-output pressure waveforms, and MAP was computed as:


$$\begin{array}{c} \begin{array}{c} MAP=DBP+\frac{SBP-DBP}{\text{3}} \end{array} \end{array}$$ (9)

The time-varying ANS modulation signal was computed as a weighted combination of ${SNS}_{i}$ and ${PNS}_{i}$:


$$\begin{array}{c} \begin{array}{c} \sigma_{i}(t)=\alpha_{i}\cdot {SNS}_{i}-\beta_{i}\cdot {PSNS}_{i}+\gamma_{i}, \end{array} \end{array}$$ (10)

where $\alpha_{i}$ and $\beta_{i}$ represent the weight levels of SNS and PSNS, respectively, and $\gamma_{i}$is the basal activation level of target organs in the absence of ANS modulation. In particular, the $\overset{\text{0}}{\underset{i}{\sigma }}$ is utilized to represent initial modulation signal at the onset of ANS engagement. The target parameters for the control loops were guided by the primary determinants of HR and BP in the CVS submodule. For HR modulation, the target parameter is the HR itself (bps), while for BP modulation, the target parameter is the total peripheral blood flow resistance (R) of the arterial tree. The modulatory dynamics of each target parameter were governed by a first-order ordinary differential process as:


$$\begin{array}{c} \begin{array}{c} \frac{dX_{i}}{dt}=\frac{-X_{i}+\sigma_{i}(t)}{\tau_{i}}, \end{array} \end{array}$$ (11)

where $X_{i}$ denotes the target parameter—HR itself for HR modulation ($X_{HR}=HR$) and R for BP modulation ($X_{BP}=R$)—and $\tau_{i}$ represents the characteristic transition time of each effector pathway in the ANS-mediated regulation process. These time constants were specified as fixed phenomenological parameters, as prior modeling studies have shown that such parameters are used to represent pathway-specific response dynamics and may vary across physiological processes and model structures [45 46]. Therefore, in the present model, $\tau_{HR}$ = 25 s and $\tau_{BP}$=5 s were adopted as fixed values to capture stable and physiologically plausible regulation dynamics within the integrated closed-loop framework. In addition to direct ANS modulation, BP modulation was also influenced by HR-driven changes in cardiac output and by respiratory effects, yielding a physiologically integrated model.

#### Autonomic nervous system functions.

Building on the autonomic regulation framework, this study characterized ANS function by separately parameterizing autonomic control modes and the weight of the SNS and PSNS. Autonomic control modes define the structural patterns of SNS–PSNS coordination that govern cardiovascular regulation, whereas weight refers to the branch-specific scaling of sympathetic and parasympathetic outputs specified at the beginning of each simulation. This formulation allows the model to distinguish between qualitative differences in autonomic regulation strategies and quantitative differences in the strength of SNS and PSNS modulation acting on different interoceptive signals.

**Fig 2:**
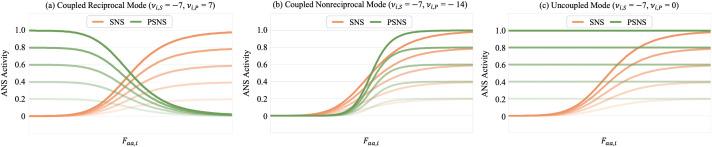
Definition of autonomic control modes and corresponding SNS–PSNS weights patterns as a function of afferent fiber activation. The model defines three autonomic control modes based on the relative interaction between SNS and PSNS branches. (a) Coupled reciprocal mode, in which SNS and PSNS exhibit opposing activation patterns with response slopes of equal magnitude but opposite sign. (b) Coupled nonreciprocal mode, in which SNS and PSNS share the same response sign, resulting in coactivation or coinhibition depending on slope direction. (c) Uncoupled mode, in which the response of one autonomic branch is withdrawn by setting its slope to zero, rendering its activity invariant across afferent input levels. Within each mode, the weight levels of SNS and PSNS are further modulated by the weighting parameters $\alpha_{i}$ and $\beta_{i}$, which scale their respective contributions to autonomic regulation.

Specifically, autonomic control modes were first systemically defined within the ANS submodule to represent distinct SNS–PSNS coordination schemes governing HR and BP regulation under different external stimuli and physio/psychological conditions. Three types of autonomic control modes were defined: coupled reciprocal, coupled nonreciprocal, and uncoupled. Fig 2 illustrates how ANS activity patterns vary with different levels of afferent fiber activation [$F_{aa,i}(t)$] under each control mode. The coupled reciprocal mode [Fig 2(a)], one of the earliest forms of autonomic control described in the previous literature, is characterized by an opposing activity between the SNS and PSNS branches, such that activation of one division is accompanied by inhibition of the other. In the model, reciprocal modes were represented by assigning opposite signs to the slope parameters $\nu_{i}$ of SNS and PSNS. Following the parameter settings in 45, the response slopes of SNS and PSNS were set to ±7 to produce responses of equal magnitude and opposite direction. In coupled nonreciprocal mode [Fig 2(b)], the $\nu_{i}$ values of SNS and PSNS were assigned the same sign: positive slopes indicate coinhibition, whereas negative slopes indicate coactivation. In the uncoupled mode [Fig 2(c)], one division was modeled by setting its $\nu_{i}$ value to zero, effectively withdrawing its response so that its activity remained constant across all $F_{aa,i}(t)$ levels. In addition to specifying control modes, the model allowed independent adjustment of the relative levels of SNS and PSNS weights using weighting parameters. Meanwhile, within each predefined control mode, the effective SNS and PSNS modulation weights could be independently adjusted at the beginning of model-based simulations, thereby determining the gain of sympathetic or parasympathetic modulation on HR and BP without altering the underlying control structure. The weighting factors $\alpha_{i}$and $\beta_{i}$ scaled the respective contributions of SNS and PSNS outputs to modulation of each target parameter. These parameters therefore functioned as effective SNS and PSNS modulation weights, controlling the relative gain of sympathetic and parasympathetic influences. To examine how variations in autonomic balance influenced HR and BP dynamics, $\alpha_{i}$ and $\beta_{i}$ were varied from 0 to 1 in increments of 0.2. As shown in Fig 2, the effective SNS and PSNS modulation weights within each control mode were systematically varied by tuning $\alpha_{i}$ and $\beta_{i}$ within this normalized range.

### Output waveforms

Fig 1(b)–1(f) illustrate representative waveforms generated by the model under closed-loop operation, including HR, BP, R, ANS outputs, and ITP. To clearly demonstrate the functional roles of the CVS, ANS, and RS modules within the integrated framework, the simulation timeline was intentionally divided into three designed stages, each highlighting the dominant contribution of a specific regulatory subsystem. These stages were defined for methodological illustration purposes rather than to represent distinct biologically occurring physiological states. The simulation was therefore structured to systematically characterize the dynamic responses of the CVS, RS, and ANS across these predefined regulatory phases. Accordingly, the time course was divided into three functionally motivated stages: (i) an initialization stage dominated by cardiovascular load, (ii) a regulation stage driven by autonomic feedback, and (iii) a modulation stage dominated by respiratory rhythms.

In Phase I (0–15 s), the model was initialized with elevated HR, BP, and R to simulate a high-load physiological state following external stimuli or internal stress. HR was set to a high level (100 bpm), and R remained elevated, reflecting significant vasoconstriction in peripheral vessels. The increases in HR and R jointly raised BP, which also exhibited cardiac cycle-synchronized oscillations. During Phase I, the ANS was inactive and no respiratory-induced ITP fluctuations were applied. This stage was designed to isolate the CVS response under a prescribed high-load condition, providing a reference state for the subsequent initiation of autonomic regulation.

In Phase II (15–65 s), the elevated HR and BP were transmitted as afferent inputs to the ANS module, initiating autonomic feedback regulation. SNS output gradually decreased, whereas PSNS output increased. Consequently, HR and R progressively declined, leading to recovery of BP toward the normal physiological range. During this process, SNS and PSNS outputs approached equilibrium, characterizing a typical coupled reciprocal pattern of ANS regulation. This phase demonstrates the role of the ANS module in restoring cardiovascular variables toward stable operating ranges through feedback control.

In Phase III (65–85 s), periodic ITP fluctuations generated by the RS module were introduced, simulating respiratory modulation of cardiac output. The rhythmic negative ITP waves induced low-frequency respiratory modulation in the BP waveform, superimposed on the higher-frequency cardiac oscillations. After ANS-mediated recovery, the mean BP level further decreased, and the ANS outputs exhibited mild fluctuations synchronized with the respiratory rhythm. This stage explicitly illustrates how respiratory dynamics, introduced after autonomic stabilization, modulate hemodynamic signals on a slower timescale.

Together, the three designed phases illustrate the dynamic behavior of the computational model across multiple regulatory timescales, with each phase emphasizing the contribution of a specific subsystem rather than depicting sequential biological stages. These simulations demonstrate the capacity of the closed-loop integration of CVS, RS, and ANS modules to reproduce key temporal characteristics of interoceptive dynamics.

## Experiment 1: HR and BP responses in TD and ASD under external stimulation

### Experimental setting

#### Model initialization.

External stimulation induces rapid shifts in ANS function, reflected in changes in autonomic control modes and SNS–PSNS weight. To evaluate autonomic regulatory capacity, HR and BP responses were simulated across different control modes and SNS–PSNS activity combinations using the computational model. The simulated responses were compared with representative empirical ranges of HR and BP reported in previous studies of TD and ASD populations, enabling identification of control modes that reproduce characteristic physiological responses under stimulation. In addition, autonomic control modes and their associated response slopes were predefined, baseline targets were specified as explicit modeling assumptions, and time constants were fixed as phenomenological parameters; thus, the resulting response surfaces should be interpreted as conditional comparative mappings under constrained parameter settings.

Previously reported HR and BP responses observed during the head-up tilt (HUT) test [47] were selected as the empirical basis for initial model validation and used to guide model configuration. The HUT test was chosen because it provides a standardized experimental paradigm for assessing autonomic regulation under gravitational perturbation in clinical research. As a passive postural challenge, HUT minimizes confounding effects from voluntary movement, skeletal muscle activation, and cognitive or emotional strategies. The test typically involves a rapid transition from a supine to an upright position (≈60–70°), inducing characteristic SNS–PSNS adjustments associated with HR elevation and BP stabilization in response to postural challenge. This standardized and relatively low-confounding setting was therefore adopted as an initial validation context for the present computational framework.

Real measured HR and BP levels before and after the HUT test (Table 1) were used to parameterize initial afferent fiber activations ($\overset{\text{0}}{\underset{aa,i}{F}}$) and baseline target levels ($\mu_{i}$) of HR and BP, respectively, for the ANS modulatory loop in the sigmoid-based framework. Median measured values at supine rest in TD and ASD groups were used to define $\overset{\text{0}}{\underset{aa,i}{F}}$ serving as the initial inputs to the model. In contrast, uniform $\mu_{i}$ values derived from the median post-HUT measurements of the TD group were applied to both groups as an explicit modeling assumption for cross-group comparison, providing a shared regulatory target intended to represent a physiologically adequate reference state under the shared physiological demands imposed by the HUT challenge, rather than a neutral physiological baseline. Full parameter settings are summarized in Table 2. These empirical quantities were therefore used to define model initialization conditions and unified reference targets, whereas the simulated response surfaces were subsequently generated by systematically varying SNS–PSNS weights within the model. Accordingly, the present framework operates at a group-summary level and was designed to provide a feasibility demonstration of the integrative computational framework under standardized representative conditions.

#### Evaluation metrics.

To evaluate ANS regulatory characteristics under different autonomic control modes, HR and BP responses were systematically simulated across combinations of $\alpha_{i}$and $\beta_{i}$within each autonomic control mode. The corresponding steady-state HR and BP outputs for each ($\alpha_{i}$, $\beta_{i}$) combination were computed, thereby generating simulated response surfaces composed of discrete simulated points. The empirical HR and BP ranges of each group were then mapped onto the corresponding simulated response surfaces to identify ($\alpha_{i}$, $\beta_{i}$) parameter points that fell within the observed ranges. Based on these points, real data distribution regions were delineated and used to compare potential autonomic regulatory patterns across groups and modes.

Coverage metrics, centroid coordinates, and three-mode Jaccard overlap were further introduced to quantify the extent, spatial location, and across-mode overlap of the real data distribution regions. The overlapping region between the SBP and DBP real data distribution regions was then defined as the final valid region for BP, as these two regions were first identified separately. If one of the two regions was empty, the other non-empty region was used as the representative BP region; if both regions were empty, the valid BP region was defined as an empty set. Accordingly, all quantitative analyses for BP were performed on this final valid region.

The coverage metrics (Cov) was defined as the proportion of parameter points falling within the empirical range relative to the total number of simulated parameter points:


$$\begin{array}{c} \begin{array}{c} Cov=\frac{\left|N_{m}\right|}{\left|N_{T}\right|},\;m\in M \end{array} \end{array}$$ (12)

where M= {coupled reciprocal, coupled nonreciprocal, uncoupled}; $N_{m}$ denotes the set of parameter points falling within the empirical range under a given control mode; and $N_{T}$denotes the total number of points on the response surface. In the present study, both $\alpha_{i}$ and $\beta_{i}$ took values from {0.2, 0.4, 0.6, 0.8, 1.0}, and therefore $N_{T}$ = 25.

To further characterize the spatial distribution of real data distribution regions across groups and control modes, the projected coordinates of the geometric centroid of each real data distribution region on the $\alpha_{i}$-$\beta_{i}$ plane, (${\overset{―}{\alpha }}_{i}$,${\overset{―}{\beta }}_{i}$), were calculated. Let H denote the number of vertices defining the boundary of real data distribution region, and let these boundary vertices be ordered as (${\overset{―}{\alpha }}_{i,\text{1}}$, ${\overset{―}{\beta }}_{i,\text{1}}$), (${\overset{―}{\alpha }}_{i,\text{2}}$, ${\overset{―}{\beta }}_{i,\text{2}}$), ⋯, (${\overset{―}{\alpha }}_{i,H}$, ${\overset{―}{\beta }}_{i,H}$), with (${\overset{―}{\alpha }}_{i,H+\text{1}}$, ${\overset{―}{\beta }}_{i,H+\text{1}}$) = (${\overset{―}{\alpha }}_{i,\text{1}}$, ${\overset{―}{\beta }}_{i,\text{1}}$). The centroid coordinates (${\overset{―}{\alpha }}_{i}$, ${\overset{―}{\beta }}_{i}$) were then defined as follows:


$$\begin{array}{c} {\overset{―}{\alpha }}_{i}=\frac{\text{1}}{\text{6}\cdot A_{d}}\sum_{h=\text{1}}^{H}\left(\alpha_{i,h}+\alpha_{i,h+\text{1}})\cdot (\alpha_{i,h}{\cdot \beta }_{i,h+\text{1}}-\alpha_{i,h+\text{1}}{\cdot \beta }_{i,h}\right),\;h\in H \end{array}$$ (13)

$$\begin{array}{c} {\overset{―}{\beta }}_{i}=\frac{\text{1}}{\text{6}\cdot A_{d}}\sum_{h=\text{1}}^{H}\left(\beta_{i,h}+\beta_{i,h+\text{1}})\cdot (\alpha_{i,h}{\cdot \beta }_{i,h+\text{1}}-\alpha_{i,h+\text{1}}{\cdot \beta }_{i,h}\right),\;h\in H \end{array}$$ (14)

$$\begin{array}{c} A_{d}=\frac{\text{1}}{\text{2}}\sum_{h=\text{1}}^{H}\left(\alpha_{i,h}{\cdot \beta }_{i,h+\text{1}}-\alpha_{i,h+\text{1}}{\cdot \beta }_{i,h}\right),\;h\in H \end{array}$$ (15)

where $A_{d}$ denotes the area of the corresponding real data distribution region. When the parameter points falling within the empirical range did not form a closed region, the regional center was defined according to the corresponding degenerate geometric case. Specifically, when fewer than three points satisfied the empirical range or when all valid points lay on a single line segment, the midpoint of the longest line segment formed by those points was taken as the centroid location; when only one point satisfied the empirical range, that point itself was taken as the centroid location.

The three-mode Jaccard overlap (J) was further calculated to quantify the degree of regional convergence across the three autonomic control modes for the same group and the same interoceptive signal, as follows:


$$\begin{array}{c} \begin{array}{c} J=\frac{\left|⋂N_{m}\right|}{\left|⋃N_{m}\right|},\;m\in M \end{array} \end{array}$$ (16)

where $⋂N_{m}$ denotes the set of parameter points that simultaneously fell within the empirical range under all three autonomic control modes, and $⋃N_{m}$ denotes the set of all parameter points that fell within the empirical range under at least one of the three autonomic control modes.

### Results

#### Autonomic regulation in TD and ASD groups.

**Fig 3:**
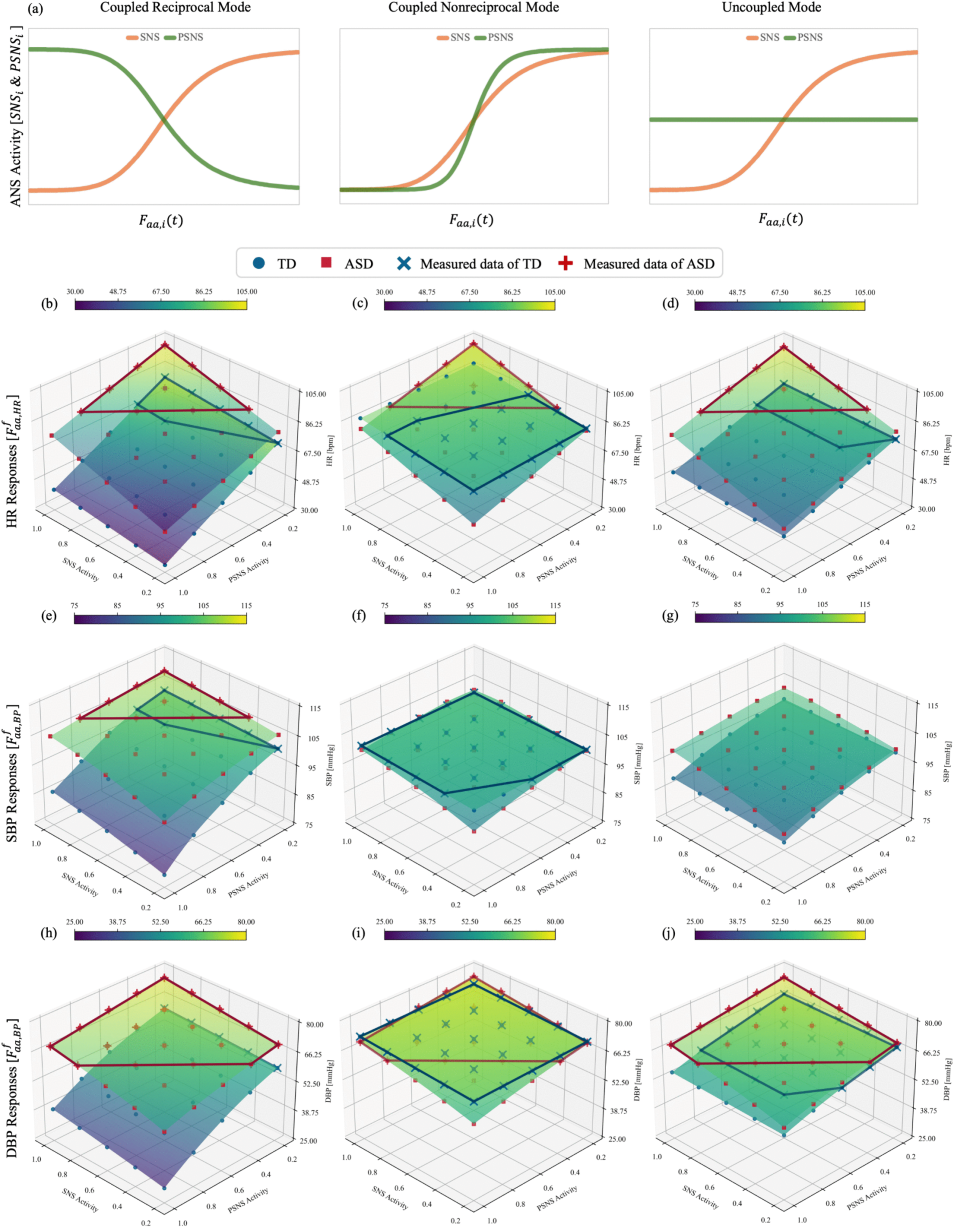
Simulated HR and BP responses of TD and ASD groups across three autonomic control modes during HUT stimulation. The upper panels illustrate the autonomic response profiles for the coupled reciprocal, coupled nonreciprocal, and uncoupled modes, generated with maximal weighting parameters for both SNS and PSNS ($\alpha_{i}$ = $\beta_{i}$ = 1.0). The three rows of 3D blue and red dotted surfaces present simulated steady-state HR, SBP, and DBP responses of TD and ASD groups, respectively, across the SNS–PSNS activity plane. Blue markers denote simulated TD responses that fall within the empirically measured HR and BP ranges, and red markers denote the corresponding ASD responses. The spatial distributions of markers across the surfaces highlight the combinations of SNS and PSNS weights for which the simulated outcomes align with empirical observations. Across the three autonomic control modes, ASD individuals exhibited higher HR, SBP, and DBP levels and greater sensitivity to changes in SNS activity compared with TD individuals, whereas TD responses aligned more closely with expected physiological patterns under the coupled reciprocal mode. In contrast, responses from the ASD group were distributed in similar SNS–PSNS regions across all three modes, indicating reduced differentiation in their autonomic regulatory behavior.

Three-dimensional (3D) surface plots were used to illustrate simulated HR and BP responses in TD and ASD groups and to examine how the computational model can help interpret latent ANS regulatory characteristics through systematic exploration of SNS–PSNS weights. Fig 3 illustrates HR and BP responses in TD and ASD groups under three autonomic control modes during HUT stimulation. A set of representative ANS activity curves shown in Fig 3(a) was generated using maximal weighting parameters for both autonomic branches, with $\alpha_{i}$ and $\beta_{i}$ fixed at 1.0. SNS and PSNS weights were systematically varied from 0 to 1.0 in increments of 0.2 to construct the response surfaces. The 3D plots in Fig 3(b)-3(j) display simulated steady-state HR, SBP and DBP responses across SNS–PSNS weights, with blue circular markers indicating TD group outcomes and red square markers indicating ASD group outcomes following HUT stimulation. Across the three autonomic control modes, the response surfaces also exhibited qualitatively different degrees of variation across SNS–PSNS weight combinations. In particular, the coupled reciprocal mode showed stronger HR and BP changes as SNS–PSNS weights varied, whereas the coupled nonreciprocal mode showed comparatively attenuated variations, with the uncoupled mode occupying an intermediate position. In addition, the blue and red cross markers on the simulated 3D surfaces indicate HR and BP responses falling within the empirically observed ranges of TD and ASD groups, while the corresponding color-coded polygons delineate the SNS–PSNS weighting regions in which simulated responses aligned with experimental measurements. These overlays provided a data-based comparative mapping to identify simulated regions consistent with the observed empirical. It is worth noting that the mapped regions should be interpreted as group-level regulatory results under representative summary-value initialization, rather than as latent regulatory properties shared by all individuals within each group. To further quantify the extent, spatial location, and across-mode convergence of these group-level regulatory landscapes, coverage, centroid coordinates of the real data distribution regions, and three-mode Jaccard overlap were additionally calculated. The corresponding results are summarized in Table 3.

On the HR response surfaces, Fig 3(b)–3(d) revealed distinct spatial distribution patterns for the TD and ASD groups across the three autonomic control modes. Under both the coupled reciprocal mode [Fig 3(b)] and the uncoupled mode [Fig 3(d)], the empirical HR data of both groups were distributed primarily within regions characterized by high SNS and low PSNS weighting. In contrast, in addition to being concentrated in the high-SNS region, the ASD group showed a further extension toward regions with relatively higher PSNS weighting under these two modes, indicating that the simulated HR responses corresponding to ASD observations were associated with comparatively higher relative PSNS weighting, suggestive of persistent parasympathetic engagement. In the coupled nonreciprocal mode [Fig 3(c)], this group difference became more pronounced: the ASD group remained clustered in regions with high SNS and low PSNS weighting, whereas the TD group shifted clearly toward regions with lower SNS and higher PSNS weighting. Thus, the spatial distributions in Fig 3 alone indicate that the TD group exhibited more prominent region shifts across modes, whereas the HR distributions of the ASD group were more similar across the three modes.

**Table 1:** Real measured heart rate (HR) and systolic and diastolic blood pressure (SBP & DBP) ranges at supine rest and HUT test conditions in TD and ASD groups.

Groups	Interoceptive signals	Supine Rest	HUT Test
TD	HR [bpm]	54.51–71.49	69.01–86.99
	SBP [mmHg]	109.51–134.19	99.49–132.51
	DBP [mmHg]	60.16–71.84	58.69–79.31
	MAP [mmHg]	76.61–92.62	72.29–97.04
ASD	HR [bpm]	62.34–91.66	82.54–115.46
	SBP [mmHg]	106.98–129.02	105.5–130.5
	DBP [mmHg]	60.63–77.37	65.89–82.11
	MAP* [mmHg]	76.08–94.59	79.09–98.24

* MAP: mean arterial pressure calculated via Eq. (9).

**Table 2:** Baseline targets ($\mu_{i}$) and afferent activations ($\overset{0}{\underset{aa,i}{F}}$, i∈{HR, BP}) for HR and BP in TD and ASD groups.

Group	$\mu_{HR}$ [bps]	$\mu_{BP}$ [mmHg]	$\overset{0}{\underset{aa,HR}{F}}$ [bps]	$\overset{0}{\underset{aa,BP}{F}}$ [mmHg]
TD	1.300	116	1.050	84.66
ASD	1.300	116	1.283	85.33

**Table 3:** Quantitative characteristics of the HR and BP real data distribution regions across three autonomic control modes in the TD and ASD groups under HUT.

Signal	Group	Autonomic control mode	Cov	${\overset{―}{\alpha }}_{i}$	${\overset{―}{\beta }}_{i}$	J
HR	TD	Coupled Reciprocal Mode	0.28	0.720	0.280	0.143
		Coupled Nonreciprocal Mode	0.68	0.467	0.629	
		Uncoupled Mode	0.36	0.648	0.295	
	ASD	Coupled Reciprocal Mode	0.40	0.800	0.400	1.000
		Coupled Nonreciprocal Mode	0.40	0.800	0.400	
		Uncoupled Mode	0.40	0.800	0.400	
BP	TD	Coupled Reciprocal Mode	0.20	0.600	0.200	0.217
		Coupled Nonreciprocal Mode	0.92	0.622	0.582	
		Uncoupled Mode	0.76	0.614	0.490	
	ASD	Coupled Reciprocal Mode	0.40	0.800	0.400	0.526
		Coupled Nonreciprocal Mode	0.76	0.678	0.522	
		Uncoupled Mode	0.76	0.678	0.522	

The quantitative results in Table 3 further reinforced this observation and more directly supported the greater potential of the coupled reciprocal mode to capture intrinsic autonomic regulatory processes in TD individuals. In the TD group, the centroid of the HR real data distribution region under the coupled reciprocal mode was located at (${\overset{―}{\alpha }}_{HR}$, ${\overset{―}{\beta }}_{HR}$)=(0.720, 0.280), corresponding clearly to a high-SNS and low-PSNS region; under the uncoupled mode, the centroid was (0.648, 0.295), which remained close to this region. In contrast, under the coupled nonreciprocal mode, the centroid shifted to (0.467, 0.629), indicating a tendency toward lower SNS and higher PSNS weighting. In addition, the three-mode Jaccard overlap for TD-HR was only 0.143, indicating that the HR parameter regions corresponding to the three modes were clearly differentiated. These results indicate that, in TD individuals, only the coupled reciprocal mode consistently anchored the empirical HR data within a high-SNS/low-PSNS region consistent with the expected physiological response to HUT, and therefore more effectively reflected typical compensatory autonomic regulation under postural challenge than the other modes. By contrast, in the ASD group, HR coverage was 0.40 under all three modes, the centroids were identical at (0.800, 0.400), and the three-mode Jaccard overlap reached 1.000, indicating that the three modes generated nearly identical real data distribution regions under the ASD-HR condition. In other words, unlike the TD group, the ASD group did not exhibit clear mode differentiation in HR regulation, but instead maintained a similar high-SNS distribution across all modes while retaining relatively higher PSNS weighting. This result not only supports the interpretation of reduced differentiation in autonomic control in ASD, but also further strengthens the inference of persistent parasympathetic engagement.

BP-based analysis revealed a pattern similar to that observed for HR, but with more pronounced differences in the width of the parameter regions and the degree of parameter constraint. Fig 3(e)–3(j) shows that, under the coupled reciprocal mode, the overlap between the SBP and DBP regions in the TD group was nearly compressed into a narrow line and was concentrated in a low-PSNS-weighting region. In contrast, under the same mode, the BP overlap region in the ASD group was not only broader in extent but also extended further toward regions with relatively higher PSNS weighting. Under the coupled nonreciprocal and uncoupled modes, the BP regions of both groups became markedly broader, with the ASD group showing a larger distributional region under both modes. In other words, in TD individuals, the BP region associated with the coupled reciprocal mode was more concentrated, more directional, and located primarily within a high-SNS/low-PSNS region. By contrast, the ASD group showed an extension toward regions with higher PSNS weighting, whereas under the coupled nonreciprocal and uncoupled modes, the BP regions of both groups became broadly distributed.

The quantitative results in Table 3 further supported the visual observations described above and strengthened the interpretation that the coupled reciprocal mode more effectively captured intrinsic autonomic regulatory processes in TD individuals. In the TD group, BP coverage under the coupled reciprocal mode was 0.20, and the centroid of the region was located at (0.600, 0.200), clearly within the high-SNS/low-PSNS region. By contrast, under the coupled nonreciprocal and uncoupled modes, coverage increased to 0.92 and 0.76, respectively, with corresponding centroids at (0.622, 0.582) and (0.614, 0.490). These results indicate that, although these modes covered a broader range of empirical BP data, their regional locations deviated substantially from the compensatory autonomic response pattern expected under HUT, namely high SNS weighting and low PSNS weighting. In addition, the three-mode Jaccard overlap for TD-BP was 0.217, further indicating that the BP parameter regions corresponding to the three modes overlapped only to a limited extent. Taken together, these results suggest that, in the TD group, although the coupled reciprocal mode yielded lower coverage, its corresponding region was more concentrated, more directional, and more consistently aligned with the high-SNS/low-PSNS region, and therefore more effectively captured the typical intrinsic autonomic regulation recruited under HUT stimulation.

In the ASD group, BP coverage under the coupled reciprocal mode was 0.40, with the centroid located at (0.800, 0.400); under the coupled nonreciprocal and uncoupled modes, coverage was 0.76 in both cases, and the centroids were identical at (0.678, 0.522). At the same time, the three-mode Jaccard overlap for ASD-BP was 0.526, which was higher than that for TD-BP, indicating that ASD individuals likewise exhibited greater across-mode convergence in BP regulation. Importantly, the higher coverage observed under the coupled nonreciprocal and uncoupled modes in TD-BP does not imply that these modes more accurately reflect autonomic regulation under HUT. Rather, this result more likely indicates weaker constraint of the parameter space by the empirical data, that is, greater degeneracy. In contrast, the smaller but more concentrated BP parameter region under the coupled reciprocal mode, located more clearly within the high-SNS/low-PSNS region, was more consistent with the compensatory autonomic response required under postural challenge. This finding indicates that the adequacy of a control mode cannot be judged solely by the magnitude of coverage, but must instead be interpreted jointly with region location and across-mode overlap.

Notably, using HR as an example, the relatively higher PSNS weighting indicated by the model for the ASD group appears inconsistent with the overall higher post-HUT HR levels observed empirically. The ASD group exhibited higher empirically measured resting HR, which was used as the initial afferent input to the model. This elevated baseline resulted in higher SNS and PSNS activation values at the onset of modulation and an upward shift of the simulated HR response surfaces. Consequently, steady-state HR after HUT remained higher in the ASD group across autonomic weighting parameters. In this study, SNS and PSNS activation values reflect instantaneous neural responses to afferent cardiovascular signals, whereas the weighting parameters α and β quantify the relative effectiveness of autonomic output. Accordingly, the elevated post-HUT HR in the ASD group is attributable to higher initial cardiovascular states, rather than more effective PSNS suppression. Importantly, ineffective PSNS suppression should not be interpreted as a relaxed physiological state, but rather as a sign of dysregulated ANS engagement under challenge.

#### Direction and magnitude of ANS regulation.

**Fig 4:**
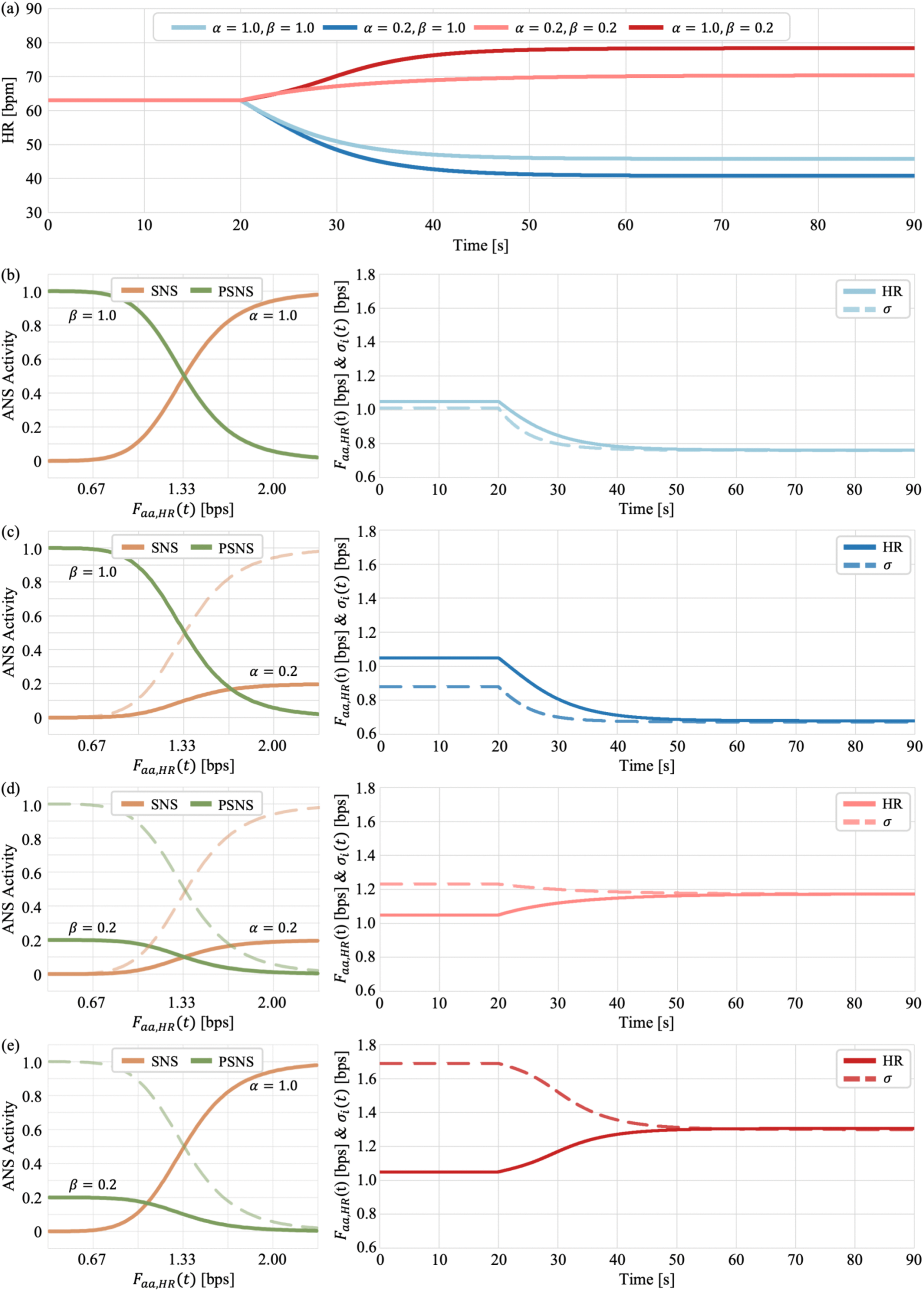
Time-dependent HR responses and underlying autonomic modulation mechanisms following the HUT test under four extreme SNS–PSNS weighting combinations (α, β) in TD individuals. (a) Simulated HR trajectories during ANS modulation for different combinations of SNS and PSNS weights. (b)–(e) Mechanistic analyses illustrating the temporal evolution of the afferent activation level of HR [$F_{aa,HR}(t)$] and the modulation signal [$\sigma_{HR}(t)$] under the corresponding weighting conditions shown in (a). When $\overset{\text{0}}{\underset{aa,HR}{F}}$ exceeds $\overset{\text{0}}{\underset{HR}{\sigma }}$ at the onset of autonomic engagement (b, c), the lower $\overset{\text{0}}{\underset{HR}{\sigma }}$ level imposes a downward regulatory drive, causing both $F_{aa,HR}(t)$ and $\sigma_{HR}(t)$ to decrease concurrently until convergence at a lower $\overset{f}{\underset{aa,HR}{F}}$. In contrast, when $\overset{\text{0}}{\underset{HR}{\sigma }}$ is initially higher than $\overset{\text{0}}{\underset{aa,HR}{F}}$ (d, e), the elevated $\overset{\text{0}}{\underset{HR}{\sigma }}$ level generates a sustained upward regulatory drive, leading to progressive HR elevation until convergence at a higher $\overset{f}{\underset{aa,HR}{F}}$level.

The general trajectories of interoceptive responses following the HUT test were evaluated under four extreme combinations of SNS and PSNS weighting parameters (α and β) to characterize the roles of these parameters in determining the direction and magnitude of ANS-modulated interoceptive regulation. As shown in Fig 4(a), ANS-modulated HR outcomes, for example, varied markedly across weighting conditions depending on the dominant autonomic branch in TD individuals. When PSNS weighting was minimal (β = 0.2), HR exhibited an upward regulatory direction. In contrast, when PSNS weighting was dominant (β = 1.0), HR showed a continuous decline toward the lower level than baseline. However, according to Eq. (11), the direction and magnitude of HR adjustments in the model were governed directly by the modulatory signal [$\sigma_{HR}(t)$], rather than by the weighting parameters themselves. Fig 4(b)–4(e) present the temporal evolution of both the HR afferent activation level [$F_{aa,HR}(t)$] and $\sigma_{HR}(t)$under the four weighting conditions. The initial HR level ($\overset{\text{0}}{\underset{aa,HR}{F}}$) was set as 1.05 bps (63 bpm) according to Table 2. In the configurations shown in Fig 4(b) and 4(c), $\overset{\text{0}}{\underset{aa,HR}{F}}$ exceeded $\overset{\text{0}}{\underset{HR}{\sigma }}$ at the onset of autonomic engagement. Under these conditions, the lower $\overset{\text{0}}{\underset{HR}{\sigma }}$ level imposed a downward regulatory influence, driving both $F_{aa,HR}(t)$ and $\sigma_{HR}(t)$ to decrease concurrently until the difference between the two signals was eliminated. HR stabilization ($\overset{f}{\underset{aa,HR}{F}}$) was achieved once $F_{aa,HR}(t)$and $\sigma_{HR}(t)$converged, resulting in a reduced post-modulation HR level. In contrast, in the configurations shown in Fig 4(d) and 4(e), $\overset{\text{0}}{\underset{HR}{\sigma }}$ was initially higher than $\overset{\text{0}}{\underset{aa,HR}{F}}$. The elevated $\overset{\text{0}}{\underset{HR}{\sigma }}$ level generated a sustained upward regulatory drive, leading to progressive HR elevation until $\sigma_{HR}(t)$ and $F_{aa,HR}(t)$ approached equivalence, and stabilization was attained. Across the weighting conditions examined in Fig 4(b)–4(e), the direction of HR variation was determined by the sign of the difference between $\overset{\text{0}}{\underset{HR}{\sigma }}$ and $\overset{\text{0}}{\underset{aa,HR}{F}}$, whereas the magnitude of HR elevation or reduction scaled with the absolute value of this difference. In these simulations, SNS and PSNS activities influenced HR responses by modulating the $\sigma_{HR}(t)$ signal, rather than acting as independent drivers of HR regulation, thereby illustrating under the fixed parameterization how different SNS–PSNS weighting conditions shape the direction and magnitude of ANS-modulated HR responses.

#### Sensitivity and uncertainty analysis.

To further examine whether the computational model depended on specific parameter settings or empirical initialization values, this section analyzed key settings affecting simulated HR responses under the coupled reciprocal mode. As described above, the coupled reciprocal mode more effectively captured the typical autonomic regulatory pattern of the TD group under postural challenge. Therefore, within this mode, we examined the effects of branch response slopes $\nu_{HR,S}$and $\nu_{HR,P}$, baseline definition, and initial afferent condition on HR responses. These analyses were conducted to assess whether the main model-based interpretations reported in this study depended on a single fixed parameter setting, or whether they remained qualitatively stable across a limited range of parameter variations and input perturbations.

We first performed a response-slope sensitivity analysis under the coupled reciprocal mode. This analysis preserved the sign structure defining the coupled reciprocal mode, in which the SNS and PSNS response slopes have opposite directions. The initial HR-related afferent activation ($\overset{\text{0}}{\underset{aa,HR}{F}}$) for the TD and ASD groups was fixed according to the initialization values shown in Table 2, and the unified baseline ($\mu_{HR}$) was also held constant. In addition, both $\alpha_{HR}$ and $\beta_{HR}$ were fixed at 1.0. Under this controlled setting, changes in the final HR responses could be primarily attributed to changes in the response slopes $\nu_{HR,S}$ and $\nu_{HR,P}$. Specifically, we varied the response slope of one autonomic branch while keeping the slope of the other branch unchanged. First, with $\nu_{HR,P}$ = 7.0 fixed, the SNS response slope $\nu_{HR,S}$ was set to −3.5, −7.0, and −14.0 to examine the effect of SNS response steepness on HR responses. Second, with $\nu_{HR,S}$ = −7.0 fixed, the PSNS response slope $\nu_{HR,P}$ was set to 3.5, 7.0, and 14.0 to examine the effect of PSNS response steepness on HR responses. Because the coupled reciprocal mode in Fig 3 was computed using a slope absolute value of 7.0, |ν| = 3.5 and |ν| = 14.0 were used to represent flatter and steeper autonomic response functions, respectively.

**Fig 5:**
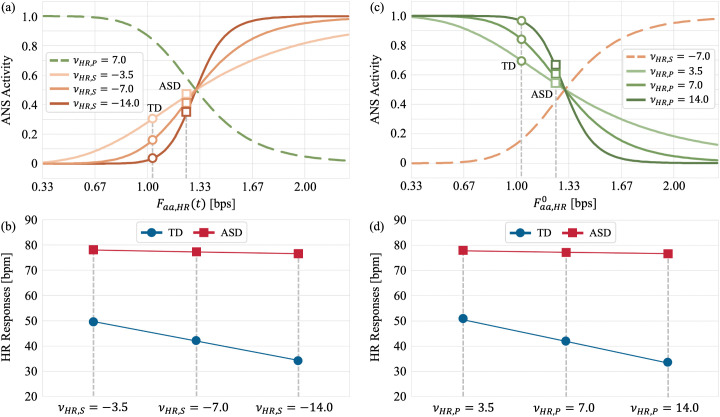
Response-slope sensitivity analysis of HR responses under the coupled reciprocal mode. (a) SNS and PSNS activity curves when the PSNS response slope was fixed at $\nu_{HR,P}$ = 7.0 and the SNS response slope $\upsilon_{HR,S}$ was varied across −3.5, −7.0, and −14.0. (b) Final HR responses of the TD and ASD groups under the corresponding $\upsilon_{HR,S}$ settings. (c) SNS and PSNS activity curves when the SNS response slope was fixed at $\nu_{HR,S}$ = −7.0 and the PSNS response slope $\nu_{HR,P}$ was varied across 3.5, 7.0, and 14.0. (d) Final HR responses of the TD and ASD groups under the corresponding $\upsilon_{HR,P}$ settings. In all simulations, the coupled reciprocal sign structure was preserved, the initial HR-related afferent activation values were fixed according to Table 2, the unified baseline was fixed at $\mu_{HR}$ = 1.3, and both $\alpha_{HR}$and $\beta_{HR}$ were set to 1.0. Changes in response slopes affected the absolute HR responses, particularly in the TD group, but ASD-HR responses remained higher than TD-HR responses across all tested slope settings.

The results shown in Fig 5 indicate that changing the response slopes affected the absolute values of the final HR responses ($\overset{f}{\underset{aa,HR}{F}}$) but did not alter the main group-level relationship in which ASD-HR responses remained higher than TD-HR responses. Whether $\upsilon_{HR,S}$ or $\upsilon_{HR,P}$ was fixed, HR responses in TD group decreased markedly as the response slopes of another branch became steeper, whereas ASD-HR responses showed only small changes. These results indicate that response-slope variation affected model outputs but did not eliminate the TD-ASD group difference. This further suggests that the TD-ASD difference reported in this study was not solely attributable to a single fixed response-slope setting but showed qualitative robustness across the tested slope settings. The difference in response-slope sensitivity patterns between the TD and ASD groups can be explained by the different locations of their initial afferent states $\overset{\text{0}}{\underset{aa,HR}{F}}$ on the sigmoid response functions. In the TD group, $\overset{\text{0}}{\underset{aa,HR}{F}}$ was in a region where changes in the response slopes substantially altered SNS or PSNS activity. Therefore, when the absolute value of $\upsilon_{HR,S}$ or $\upsilon_{HR,P}$ was changed, both the estimated ANS activity and the final HR responses showed relatively large changes. In contrast, in the ASD group, $\overset{\text{0}}{\underset{aa,HR}{F}}$ was closer to the unified baseline $\mu_{HR}$ = 1.3. Under the current slope manipulation conditions, the corresponding SNS or PSNS activity changed less markedly, and the final HR responses therefore showed lower sensitivity to response-slope variation.

**Fig 6:**
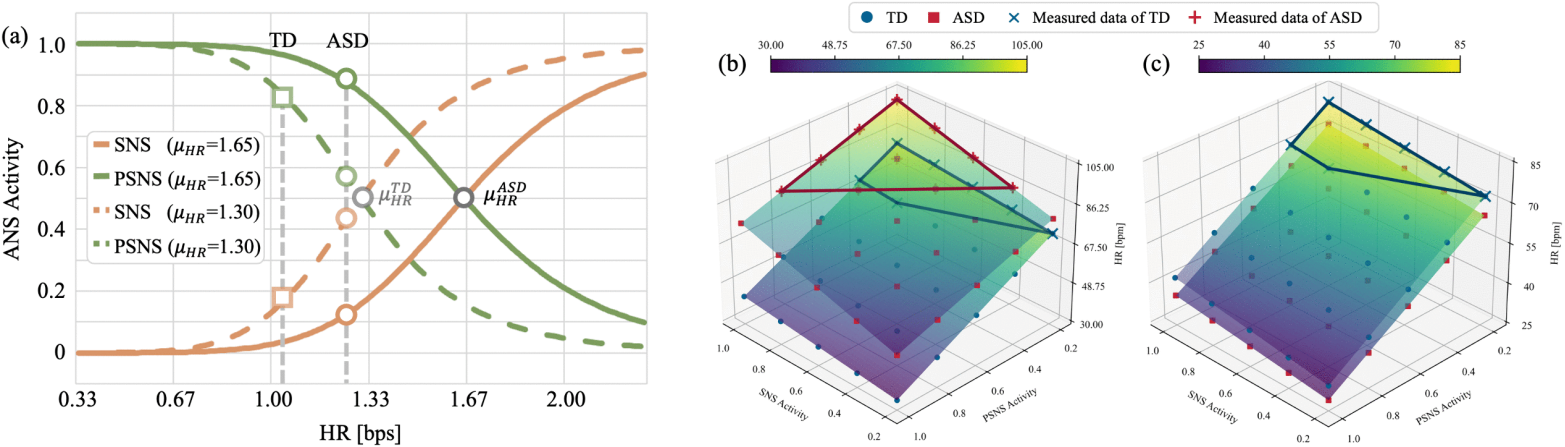
Effects of baseline selection on autonomic activity estimates and simulated HR responses. (a) Sympathetic (SNS) and parasympathetic (PSNS) activities derived from the sigmoid-based autonomic modulation function under two baseline settings: $\mu_{HR}$= 1.30 bps (78 bpm; solid lines) and $\mu_{HR}$ = 1.65 bps (99 bpm; dashed lines). The TD resting HR (63 bpm) and ASD resting HR (77 bpm) produce distinct $\overset{\text{0}}{\underset{aa,HR}{F}}$ values, which yield clearly differentiated SNS–PSNS balances when a unified baseline is applied. Assigning the ASD post-HUT HR (99 bpm) as a group-specific baseline shifts the predicted autonomic state toward a falsely normalized pattern and masks characteristic ASD autonomic imbalance. (b) Simulated HR surfaces across SNS–PSNS weighting combinations under the unified baseline ($\mu_{HR}$= 1.30 bps), overlaid with measured HR values from TD and ASD individuals following the HUT test. The TD data cluster aligns with the physiologically expected high-SNS/low-PSNS region, whereas ASD data extend toward elevated PSNS weight. (c) Simulated HR surfaces generated using the ASD-specific elevated baseline ($\mu_{HR}$= 1.65 bps). The increased baseline compresses the effective regulatory range, resulting in attenuated HR responses that fail to capture empirical ASD data distribution patterns, thereby illustrating how an abnormally high baseline distorts simulated autonomic trajectories.

In addition to response slopes, baseline definition is also a key modeling setting that affects the simulated results. To evaluate how baseline selection influences model behavior and interpretation, this study compared a unified baseline definition against an alternative group-specific baseline setting based on empirically measured post-HUT interoceptive signals. Fig 6 illustrates the baseline HR selection under coupled reciprocal mode and simulated HR responses. The unified baseline $\mu_{HR}$ = 1.300 bps (78 bpm) effectively reveals marked differences in the initial regulatory state [Fig 6(a)] within the SNS and PSNS activation levels [Eqs. (7) and (8)]. The TD group exhibited an $\overset{\text{0}}{\underset{aa,HR}{F}}$of 1.050 bps (63 bpm), which was associated with relatively low SNS weighting and high PSNS weighting (SNS = 0.183, PSNS = 0.817). In contrast, the ASD group exhibited an $\overset{\text{0}}{\underset{aa,HR}{F}}$ of 1.283 bps (77 bpm), and under the same baseline, showed substantially elevated SNS activity and reduced PSNS activity (SNS = 0.477, PSNS = 0.523), reflecting a characteristic pattern of autonomic imbalance. Assigning the ASD post-HUT HR value of 1.650 bps (99 bpm) as a group-specific baseline would lead the model to interpret this elevated HR as the intended regulatory target. Under this condition, the resulting SNS and PSNS estimates (SNS = 0.147, PSNS = 0.853) would fall within an apparently typical range, thereby obscuring abnormalities in ASD autonomic regulation. When $\mu_{HR}$ is set to 99 bpm, the simulated ASD HR response under the coupled reciprocal mode becomes lower than that of the TD group, and no empirical ASD HR values fall within the corresponding response surface. Elevated baseline values reduce initial sympathetic drive and prematurely enhance parasympathetic activation, leading to a markedly restricted regulatory range. Baseline values anchored to abnormally elevated physiological states therefore lack biological validity and distort simulated regulatory trajectories by artificially normalizing characteristic autonomic features in ASD. Such baseline adjustment would further compromise comparability between groups by eliminating a shared regulatory target, making it impossible to evaluate regulatory deviation or efficiency across conditions. Together, these comparisons indicate that baseline selection is methodologically consequential, directly affecting sigmoid activation, effective regulatory range, and the resulting interpretation of autonomic balance within the model; accordingly, the unified TD-derived post-HUT baseline should be understood as a modeling assumption introduced to preserve a shared regulatory reference across groups, rather than as a neutral baseline choice.

To examine whether simulated HR responses depended on the precise initialization value without introducing additional distributional assumptions, we further performed a ± 5% perturbation analysis of $\overset{\text{0}}{\underset{aa,HR}{F}}$ using HR responses as an example. This analysis was conducted to test whether local uncertainty in HR-related empirical initialization affected the simulated results. $\overset{\text{0}}{\underset{aa,HR}{F}}$ was selected as the perturbation target because this parameter was directly derived from empirical group-summary initialization and was entered into the ANS modulation loop as HR-related afferent activation. Therefore, this analysis allowed us to examine how empirical input uncertainty propagated to simulated HR responses. We fixed either $\alpha_{HR}$ = 1.0 or $\beta_{HR}$ = 1.0, while varying the other branch weight from 0.2 to 1.0, to evaluate whether perturbations in $\overset{\text{0}}{\underset{aa,HR}{F}}$ altered the branch-specific HR response profiles.

**Fig 7:**
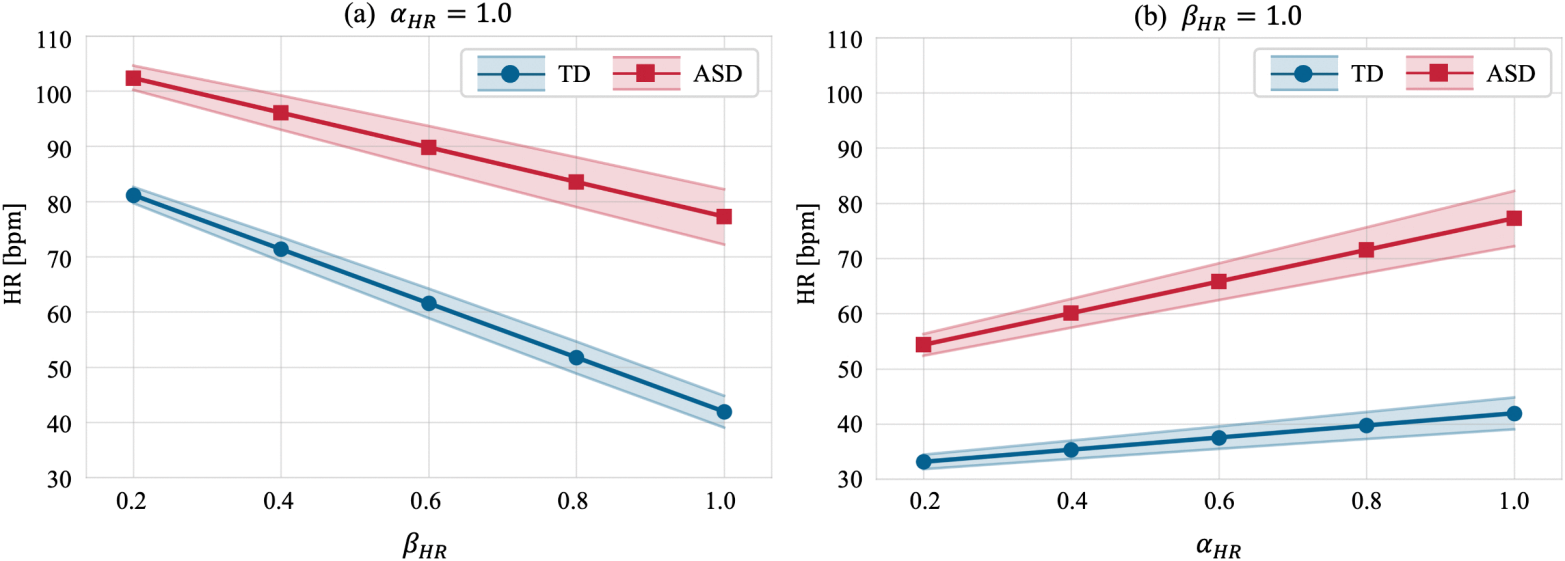
Local uncertainty analysis of HR responses under the coupled reciprocal mode. The initial HR-related afferent activation $\overset{\text{0}}{\underset{aa,HR}{F}}$ was perturbed by ±5% to examine whether local uncertainty in empirical initialization affected branch-specific HR response profiles. Shaded regions indicate the output ranges generated by ±5% perturbation of $\overset{\text{0}}{\underset{aa,HR}{F}}$, and solid lines indicate HR responses under the original initialization values. (a) HR responses when $\alpha_{HR}$ = 1.0 was fixed and $\beta_{HR}$ was varied from 0.2 to 1.0. Increasing $\beta_{HR}$ progressively reduced HR responses in both TD and ASD groups. (b) HR responses when $\beta_{HR}$ = 1.0 was fixed and $\alpha_{HR}$ was varied from 0.2 to 1.0. Increasing $\alpha_{HR}$ progressively elevated HR responses in both groups. Across both branch-specific profiles, ASD-HR responses remained higher than TD-HR responses, indicating that the main group-level relationship and branch-specific response directions were preserved under local perturbation of $\overset{\text{0}}{\underset{aa,HR}{F}}$.

The results shown in Fig 7 indicate that the ± 5% perturbation of $\overset{\text{0}}{\underset{aa,HR}{F}}$ changed the absolute range of HR responses, but did not alter the main directional trends. When $\alpha_{HR}$ = 1.0, HR responses in both the TD and ASD groups decreased consistently as $\beta_{HR}$ = 1.0 increased, indicating that increasing PSNS weight produced a sustained HR-lowering effect under an SNS-dominant condition. When $\beta_{HR}$ = 1.0, HR responses in both groups increased consistently as $\alpha_{HR}$ increased, indicating that increasing SNS weight produced a sustained HR-elevating effect under a PSNS-dominant condition. These two branch-specific response directions were not reversed after $\overset{\text{0}}{\underset{aa,HR}{F}}$ was increased or decreased by 5%. At the same time, ASD-HR responses remained higher than TD-HR responses across all branch-weight conditions, indicating that the overall upward displacement of ASD-HR responses was preserved under local perturbation of $\overset{\text{0}}{\underset{aa,HR}{F}}$. In other words, local perturbation of empirical initialization affected the absolute level of simulated HR responses, but did not change the directionality of SNS/PSNS branch-specific modulation under the coupled reciprocal mode or the main relative relationship between the TD and ASD groups. These results suggest that this HR response profile showed qualitative robustness to local uncertainty in $\overset{\text{0}}{\underset{aa,HR}{F}}$.

Further examination of the branch-specific profiles revealed different response patterns to SNS and PSNS weight changes between the TD and ASD groups. When $\alpha_{HR}$ = 1.0 was fixed and $\beta_{HR}$ was increased, TD-HR responses showed a larger decrease than ASD-HR responses, suggesting that the TD group was more sensitive to PSNS-dependent HR reduction in this model profile. Conversely, when $\beta_{HR}$ = 1.0 was fixed and $\alpha_{HR}$ was increased, ASD-HR responses showed a larger increase than TD-HR responses, suggesting that the ASD group was more sensitive to SNS-dependent HR elevation. This pattern may reflect the higher initial afferent activation in the ASD group under the unified baseline, which shifted the response profile upward and produced a larger output uncertainty range in response to the same magnitude of input perturbation. This result further supports the interpretation that ASD autonomic regulation may be associated with elevated HR responses and altered branch-specific modulation.

Overall, TD individuals exhibited clearly differentiated SNS–PSNS weight patterns across autonomic control modes, enabling identification of a dominant regulatory strategy recruited in response to external postural challenge. In contrast, ASD individuals showed greater similarity in SNS–PSNS activity distributions across the three control modes, suggesting reduced differentiation in autonomic control. This convergence implies that, when exposed to the same external stimulus, ASD individuals may not consistently engage a stable or mode-specific ANS regulatory strategy to modulate interoceptive signals. The coupled reciprocal mode demonstrated greater potential to capture intrinsic autonomic regulatory processes in TD individuals. Under this mode, empirical HR and BP values in the TD group were primarily distributed within regions characterized by high SNS and low PSNS weightings, which is consistent with the expected physiological response to HUT stimulation. In contrast, although most ASD observations were likewise located within regions of elevated SNS weighting, their distributions extended toward areas consistent with ineffective PSNS suppression (higher PSNS weighting). This pattern suggests persistent parasympathetic engagement following HUT stimulation, which may constrain ANS-mediated interoceptive adjustment and thereby reduce the adaptability of cardiovascular regulation to external perturbations. At the same time, the results of the sensitivity and uncertainty analyses further support the interpretation that the main model-based findings were not determined solely by a single response-slope setting, baseline definition, or precise empirical initialization value, but showed qualitative robustness within the tested range.

## Experiment 2: Respiratory effects on ANS-modulated BP restoration

### Experimental setting

The present study also investigated the modulatory impact of respiration on ANS-regulated BP restoration process. The model was initialized at a relatively high BP level, reflecting a physiological condition in which blood pressure has already been elevated by internal variability or prior external challenges. The MAP level [Eq. (9)] were selected as key metrics and systematically examined under varying combinations of SNS–PSNS weight weights. Physiologically, once the internal or external challenges are relieved, elevated BP is typically reduced through baroreflex-mediated autonomic regulation. This recovery process is known to involve a coupled reciprocal mode [29–31], facilitating the gradual restoration of BP toward normal physiological levels. Accordingly, respiratory effects on BP restoration were examined under the coupled reciprocal mode, in which sympathetic activity is progressively suppressed while parasympathetic activity is concurrently enhanced.

**Fig 8:**
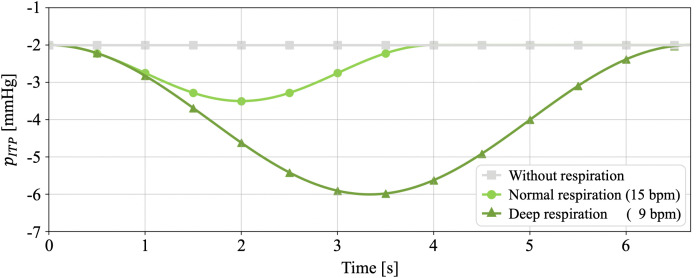
Single-cycle intrathoracic pressure (ITP) waveforms under three respiratory conditions during BP restoration. Respiratory-related ITP fluctuations are shown for (1) without respiration, (2) normal respiration characterized by low amplitude and high frequency (15 bpm), and (3) deep respiration characterized by higher amplitude and lower frequency (9 bpm).

The model was further examined under three respiratory conditions during BP restoration [Fig 8]: (1) without respiratory influence; (2) under a normal respiratory pattern, characterized by relatively low amplitude and high frequency (15 bpm); and (3) under a deep respiratory pattern, characterized by higher amplitude and lower frequency (9 bpm). Under this setting, respiratory-related ITP fluctuations [Eq. (6)] followed the patterns summarized in Table 4.

**Table 4:** Parameter settings for ITP fluctuations, $p_{ITP}(t)$, under different respiratory conditions.

Conditions	$a_{0}$ [mmHg]	a [mmHg]	ω [rad/s]
Normal respiration	−2.75	0.75	1.57 [15 bpm]
Deep respiration	−3.00	2.00	0.94 [9 bpm]
Without respiration *	−2.00	0	0

* For the “Without respiration”, a constant baseline offset (−2.00 mmHg) was retained to represent a static ITP level in the absence of respiratory oscillations.

### Results

**Fig 9:**
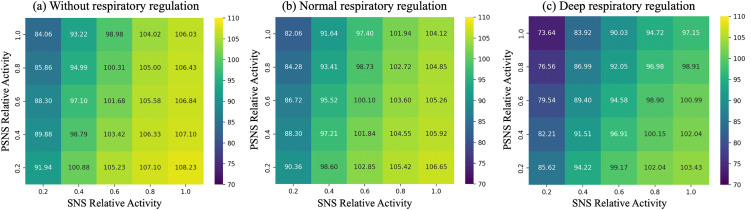
Respiratory modulation of ANS-mediated MAP restoration across SNS–PSNS activity space. Stabilized BP ($\overset{f}{\underset{aa,BP}{F}}$) following ANS modulation across combinations of SNS-PSNS weight (0–1) under (a) absence of respiratory modulation, (b) normal respiration, and (c) deep respiration. Normal respiration produced MAP levels comparable to those without respiratory influence, whereas deep respiration resulted in an overall downward shift in MAP. Under conditions of high SNS activity, increasing PSNS weighting was associated with a more pronounced MAP reduction under deep respiration than under the other respiratory conditions.

Fig 9 shows the stabilized BP levels ($\overset{f}{\underset{aa,BP}{F}}$) after ANS modulation across combinations of SNS and PSNS relative weights, with α and β ranging from 0 to 1. Under normal respiration [Fig 8(b)], the $\overset{f}{\underset{aa,BP}{F}}$was comparable to that observed in the absence of respiratory influence [Fig 9(a)], whereas deep breathing resulted in an overall lower MAP level [Fig 9(c)]. For instance, when both SNS and PSNS activities were set to 0.6, $\overset{f}{\underset{aa,BP}{F}}$ values were similar between the no-respiration (101.68 mmHg) and normal breathing (100.10 mmHg) conditions, whereas a marked reduction was observed under deep breathing (94.58 mmHg). This pattern indicates that respiratory activity can further increase the magnitude of BP reduction during ANS-mediated restoration, with larger ITP amplitudes and longer oscillation periods, such as those observed during deep respiration, being associated with lower $\overset{f}{\underset{aa,BP}{F}}$ levels.

Under SNS hyperactivity (e.g., SNS = 1.0), distinct BP regulation patterns emerged depending on respiratory modulation. As PSNS weight increased from 0.2 to 1.0, a clear decreasing trend in $\overset{f}{\underset{aa,BP}{F}}$ was observed under the deep respiratory regulation, whereas the reductions were much smaller in the absence of respiratory influence and under normal breathing. Specifically, the $\overset{f}{\underset{aa,BP}{F}}$ decreased by 2.20 mmHg without respiratory input, by 2.53 mmHg under normal respiration, and by 6.28 mmHg under deep respiration. The stepwise decline demonstrates that deep respiratory modulation enhances both the sensitivity and efficacy of PSNS regulation, thereby increasing the extent of BP recovery.

The observed improvement in regulatory performance may be associated with low-frequency ITP oscillations introduced by deep respiration. These oscillations can repeatedly shift the pressure signal into the high-slope region of the baroreflex sigmoid response function, particularly under conditions of elevated PSNS weighting. Increased gain within this region may amplify the PSNS contribution to the overall control signal $\sigma_{i}$, resulting in a greater reduction in BP. However, because the present respiratory module represents breathing effects only through periodic ITP modulation and does not explicitly implement direct bidirectional ANS–respiratory coupling, this interpretation remains limited to a model-consistent phenomenological explanation. Therefore, the physiological mechanisms underlying the deep-breathing effect require further empirical and modeling validation.

## Discussion

Although previous experimental and computational researches have investigated multiple system interactions in the human body, autonomic regulatory mechanisms underlying interoceptive modulation—and their relationships with neurodevelopmental conditions—have not been explicitly quantified or systematically characterized. Within the proposed modeling framework, this study defined ANS functions as autonomic control modes and weights of each ANS branch. Differences of ANS regulation between TD and ASD individuals were also estimated via computational model integrating multiple physiological models.

### Estimation of ANS functions in TD and ASD individuals

Empirical data in the TD group were primarily distributed within regions characterized by high SNS and low PSNS weight, which is consistent with the expected physiological response to HUT stimulation. Under quantification analysis of empirical data distribution in the TD group, the coupled nonreciprocal mode showed higher coverage than the coupled reciprocal mode for both HR and BP, indicating that a larger number of parameter combinations fell within the empirical ranges under the nonreciprocal mode. However, this larger coverage primarily reflects greater parameter feasibility, that is, a broader admissible region in parameter space through which the model can accommodate the empirical data; it does not necessarily indicate that this mode more faithfully captures the underlying physiological regulatory mechanism under HUT. Under postural challenge, the more critical issue is whether the parameter region corresponding to the empirical data is stably located within a physiologically meaningful regulatory region, namely one characterized by high SNS and low PSNS weights. From this perspective, although the coupled reciprocal mode yielded lower coverage, it more consistently anchored the responses within the region expected for compensatory autonomic regulation under HUT, thereby demonstrating stronger mechanistic alignment.

Compared with TD individuals, this study indicated the ASD group was predominantly distributed in regions characterized by concurrently high SNS and high PSNS weights across all three autonomic control modes. This pattern indicates that PSNS weights in the ASD group was not comparably reduced under external stress, suggesting ineffective parasympathetic suppression in response to external demands and potentially constraining effective modulation of physiological signals and subsequent behavioral regulation. Muscatello et al. [48] reported blunted PSNS responses in older children with ASD during peer interaction tasks, which were associated with altered stress responsivity and behavioral regulation. Similarly, Ward et al. [49] observed that higher parasympathetic influence was positively associated with ADHD symptom severity during short-term memory storage, suggesting that elevated or insufficiently modulated PSNS activity may contribute to reduced responsiveness to external cognitive or environmental demands. The response-slope sensitivity analysis provides an additional model-based indication of this regulatory inflexibility from the perspective of autonomic response sensitivity. Interoceptive responses in TD group were more strongly affected by changes in response slopes influenced by individual differences, physiological state, task context, and other factors not fully specified. However, responses in the ASD group showed smaller slope-dependent changes and elevated across the tested slope settings. Within the present modeling framework, this pattern may be consistent with a broader adjustable range of body signals in the TD group in response to changes in autonomic response sensitivity. In contrast, the reduced sensitivity of regulation to slope variation in ASD, together with their consistently elevated interoceptive levels, may indicate a more restricted regulatory range under the same shared target. The present model may offer a computational account of the prior findings by characterizing autonomic regulation including but not limited to branch-specific weights or response slopes, thereby providing an interpretable framework for understanding blunted ANS functions across different neurodevelopmental disorders.

In addition, the present findings indicate ASD individuals exhibit pronounced instability in autonomic regulation, as convergent SNS–PSNS weighting patterns render multiple autonomic control modes similarly capable of accounting for their ANS responses under identical external stimulation. ANS instability is associated with elevated interoceptive prediction errors transmitted to higher-order neural systems, as emphasized in theoretical accounts of interoceptive inference [19]. Such elevated prediction errors have been proposed to contribute to cognitive experiences marked by reduced confidence, diminished clarity, and a weakened sense of reality [20]. ANS instability reduces strategy specificity and limits the brain’s ability to correctly attribute bodily changes to internal or external causes [21]. This impaired attribution may further disrupt the integration of bodily signals with perceptual and contextual information, thereby destabilizing subjective experience, including emotional regulation and cognitive processes. Taken together, the observed autonomic instability, reduced interoceptive precision, elevated prediction error, and impaired attribution delineate a mechanistic sequence linking altered autonomic regulation to changes in cognitive experience. This physiological-to-cognitive framework provides a coherent interpretation of the empirical findings and supports the view that atypical autonomic dynamics may contribute to differences in perceptual and experiential processing in ASD.

We also systematically analyzed the HR trajectories as an example to discuss the determinants of variation directions and magnitudes of ANS-modulated interoceptive signals. Within the ANS regulatory framework, directions and magnitudes of interoceptive variation were determined by the sign and the absolute value of difference between baseline level and initial level. However, under specific combinations of SNS-PSNS weight, HR failed to exhibit a compensatory increase following the HUT stimulus and instead showed a further reduction, deviating from the typical physiological response observed in healthy individuals after postural transition. For example, the model predicted that ANS-modulated HR stabilized at a level lower than the regulatory target μ when both α and β were set to 1.0 [Fig 4]. It is worth noting that HR-suppressive responses emerging under high PSNS gain conditions within the model are not entirely absent in physiology, which delineate a potential pathological ANS regulatory phenotype. Multiple HUT studies in patients with vasovagal syncope have reported that, in a subset of individuals, HUT elicits abnormal cardioinhibitory response patterns characterized by abnormally enhanced PSNS activity and pronounced HR suppression [50–51]. In severe cases, this pathological phenotype can lead to marked bradycardia or transient cardiac asystole [52]. Accordingly, within a clinical context, HR decreases following HUT are generally interpreted as a manifestation of ANS dysregulation rather than normal compensatory regulation. The empirical HR distribution of the TD group on the simulated response surface [Fig 3] was concentrated in regions with high SNS and low PSNS weightings, where the model produced post-HUT HR elevations consistent with compensatory regulation. Although HR-suppressive patterns emerged in other SNS-PSNS weight regions, these did not overlap with the empirical HR of TD group. Overall, these findings demonstrate that the present model captures ANS regulatory patterns consistent with physiological expectations under healthy conditions while also explicitly identifying abnormal regulatory behaviors associated with ANS dysfunction within the broader parameter space. This capacity provides a structured computational framework for interpreting interoceptive response mechanisms across distinct ANS regulatory states.

This study implemented unified baseline interoceptive signals as a shared autonomic target for cross-group comparison, rather than treating baseline definition as a neutral modeling choice. Within the present framework, the unified baseline was intended to represent a physiologically adequate regulatory reference under HUT, against which regulatory deviation and efficiency in TD and ASD could be compared. Importantly, baseline interoceptive levels in the present framework represent ideal regulatory targets following stimulation, yet these targets may themselves be progressively elevated by sustained physiological or psychological stress. This interpretation aligns with allostatic load (AL) theory, which describes the cumulative physiological burden generated by repeated or chronic regulatory activation. Chronically elevated interoceptive states, such as persistently increased HR, may be incorporated into autonomic reference values, shifting the internal regulatory set point upward. Baseline-based sensitivity analysis [Fig 6] illustrate that excessively elevated baselines restrict the effective regulatory range and yield blunted HR responses that lack typical compensatory dynamics. Although such set point shifts may support short-term stability, they reduce system sensitivity and limit effective autonomic compensation. Meanwhile, elevated allostatic load has been shown to be associated with reduced physical functioning, affective disturbances, and increased cognitive vulnerability [53–54]. Accordingly, the unified baseline approach serves not only as a methodological requirement for TD–ASD comparisons, but also as a physiologically grounded framework for interpreting how sustained physiological burden may shape atypical ANS regulation and its cognitive implications.

### Functional capabilities of the computational model

Previous ANS regulation models based on baroreceptor feedback designate BP as the primary regulatory target [44–46], while HR modulation is treated as a secondary mechanism to stabilize BP. Although HR components may be included structurally in such models, parameterization and optimization typically remain BP-centered, lacking a systematic framework in which HR is modeled as an independent output variable. This limitation reduces explanatory power when applied to physiological or psychological conditions characterized by asynchronous HR and BP responses, including disease states, autonomic dysfunction, or neurodivergent phenotypes. For example, Fukuda et al. [30] and Kollai et al. [31] reported that under severe hypoxia, both HR and BP increase to enhance oxygen exchange efficiency, whereas under mild hypoxia, BP increases while HR often remains stable, preserving hemodynamic balance. Differences in modulatory mechanisms between severe and mild hypoxia indicate that HR and BP are not always regulated synchronously and that the ANS may adopt differentiated control strategies depending on the physiological context. The present computational model enables independent modulation of HR and BP, which is therefore physiologically justified and a capability not supported by previous baroreflex-based models.

Importantly, the responses of the ANS branches ($n_{s}$ and $n_{p}$) and the effective SNS and PSNS modulation weights (α and β) play distinct yet complementary roles. The response levels of the SNS and PSNS represent the immediate activation of the ANS in response to incoming interoceptive signals (current HR and BP levels), whereas the weighting parameters α and β scale the peripheral effectiveness of SNS and PSNS outputs on target organs, including the heart and blood vessels. Accordingly, α and β should be interpreted as inferred model parameters capturing effective modulation weights, rather than as direct physiological measurements of sympathetic or parasympathetic branch activity. ANS function is typically inferred from aggregate cardiovascular outputs in experimental or clinical settings, whereas internal regulatory components cannot be independently isolated or systematically manipulated. By contrast, the present model allows controlled variation of parameters associated with distinct regulatory levels, enabling separate examination of neural activation patterns and their modulation effectiveness in cardiovascular regulation. This structured decomposition therefore provides a mechanistic framework for exploring how combinations of autonomic responsiveness and modulation efficacy contribute to interoceptive dynamics, thereby facilitating more detailed comparisons of autonomic regulation between TD and ASD individuals than is possible using empirical measurements alone.

The present framework also enables controlled examination of how specific regulatory components shape simulated cardiovascular responses. In addition to varying autonomic control modes and SNS–PSNS weights, the sensitivity and uncertainty analyses in this study further demonstrate that response slopes, baseline definitions, and empirical initialization values can be selectively manipulated within the computational model. This capability allows the model to isolate how autonomic responsiveness, regulatory targets, and initial afferent states contribute to interoceptive response profiles. In this sense, the framework provides an interpretable computational platform for examining how distinct regulatory assumptions influence inferred autonomic patterns in different neurodevelopmental conditions.

In addition to simulating HR and BP elevations in response to external stimulation, the proposed model can also predict recovery trajectories following stimulus cessation. As a practical application, a respiratory module was introduced to examine how distinct breathing patterns modulate BP recovery. This module simulates normal and deep breathing by adjusting the frequency and amplitude of ITP fluctuations, manifesting in the BP waveform as lower-frequency, longer-period components relative to the cardiac cycle. Model simulations indicated that, in the absence of breathing and normal breathing, elevated PSNS activity alone was insufficient to induce substantial BP reduction when SNS activity remained high. In contrast, when deep breathing at a rate of 9 bpm was introduced, PSNS contributions to BP recovery were markedly enhanced even under elevated SNS tone, suggesting that deep breathing facilitates PSNS-driven modulation. Consistent with these findings, prior studies have shown that resonance-frequency breathing reduces blood pressure, enhances heart rate variability, and shifts autonomic balance toward increased PSNS and reduced SNS activity, alongside improvements in stress, anxiety, and cognitive function [55–56].

### Limitations and future work

The present study has several limitations. First, the computational model explicitly distinguishes multiple regulatory parameters across different functional levels, and the present study explored a selected subset of the parameter space through response-slope sensitivity analysis, comparison of unified and group-specific baseline definitions, and local perturbation of empirical initialization. However, these analyses were conducted as targeted checks under selected parameter settings, mainly to assess the quantitative stability of the modeling framework and the robustness of the simulated results within the tested ranges. In addition, fixed characteristic time constants were adopted to reduce parameter dimensionality and to isolate the effects of autonomic control modes and relative branch weighting, while maintaining physiological plausibility and numerical stability within the closed-loop framework. However, time constants in baroreflex-related dynamics may vary across individuals, tasks, and physiological conditions. Second, because HR and BP are jointly shaped by partially overlapping regulatory mechanisms in the integrated model, the present study did not fully characterize how HR-related and BP-related regulatory pathways interact across the broader autonomic parameter space. Third, the single empirical dataset from a prior HUT study was used to define initialization conditions and shared reference targets and was subsequently compared with simulated response surfaces. The findings in this study, therefore, should be interpreted as data-based and group-level explanations. Finally, although the model suggested that respiratory-related modulation may contribute to BP restoration, the respiratory module was implemented as a phenomenological periodic ITP input and does not include direct bidirectional coupling between the ANS and respiratory systems. Thus, the present model can capture respiration-related modulation of cardiovascular dynamics, but it cannot fully explain the neural feedback interactions through which respiration and autonomic outflow co-regulate each other over time.

Future work should address these limitations in several directions. First, broader parameter combinations should be examined to conduct more comprehensive sensitivity and uncertainty analyses, including systematic evaluation of response slopes, baseline definitions, time constants, weighting parameters, and HR–BP pathway interactions. Such analyses would clarify how baseline shifts, autonomic response sensitivity, branch weighting, and time-dependent regulation jointly shape interoceptive regulation. Second, the framework should be evaluated using independent cohorts and additional experimental paradigms, including distinct physiological and psychological challenge conditions. These extensions would help determine whether the regulatory patterns observed here generalize across populations, task contexts, and physiological conditions. Third, incorporating subject-specific initialization and individual-level parameter estimation would allow the model to better capture inter-individual and within-group heterogeneity, particularly the pronounced variability expected in ASD and other neurodivergent populations. Finally, future models should incorporate more explicit respiratory–ANS interactions and additional interoceptive channels, such as cardiovascular, respiratory, electrodermal, and gastrointestinal dynamics. These developments would support a more comprehensive multimodal simulation platform for ANS function assessment, interoceptive regulatory mechanism analysis, and physiologically grounded intervention prediction across a wider range of neurodivergent populations.

## Conclusions

This study developed a closed-loop computational framework integrating the CVS, RS, and ANS to systematically estimate autonomic regulatory functions underlying interoceptive modulation in TD and ASD individuals. By explicitly parameterizing autonomic control modes and branch-specific relative weights, the framework enables quantitative comparison of autonomic regulatory strategies across neurodevelopmental conditions, extending beyond descriptive characterization of autonomic dysfunction. HR and BP responses to the head-up tilt (HUT) test were simulated, and regulatory surfaces were compared with experimental HR and BP data from TD and ASD groups. TD groups exhibited differentiated SNS–PSNS coordination patterns across control modes, whereas ASD individuals demonstrated a greater convergence of SNS–PSNS weights, suggesting reduced flexibility and potential instability in autonomic control strategies under the same stimulus. Analysis of empirical HR and BP distributions on simulated responses surfaces following the HUT test indicates that the coupled reciprocal control mode demonstrates greater capacity to capture intrinsic autonomic regulatory processes in TD individuals. Under this mode, simulated SNS–PSNS weight patterns aligned most closely with expected physiological responses to postural challenge. In contrast, although the majority of ASD observations were likewise located within regions of elevated SNS weighting, empirical data distribution extended toward areas associated with relatively higher PSNS weighting on the simulated regulatory surfaces. This distributional shift suggests persistent parasympathetic engagement under postural challenge, which may limit effective ANS-mediated interoceptive adjustment in ASD, thereby reducing the adaptability of cardiovascular regulation under external stimulation. Simulations under the coupled reciprocal mode were performed under normal respiration, deep respiration, and absence of respiration to evaluate their effects on mean arterial pressure (MAP) across varying SNS–PSNS activity combinations. Incorporation of deep respiration enhanced PSNS effectiveness in lowering MAP during BP recovery, particularly under conditions of over-activity of SNS. Collectively, these findings support a mechanistic, state-space framework for characterizing autonomic coordination by linking measurable interoceptive signals to their underlying control architectures. By enabling quantitative inference of regulatory stability in TD and ASD populations and identifying respiration as a model-based modulatory factor in cardiovascular recovery, the proposed approach provides an extensible computational strategy for probing autonomic regulation across neurodevelopmental conditions.
